# Drug binding dynamics of the dimeric SARS-CoV-2 main protease, determined by molecular dynamics simulation

**DOI:** 10.1038/s41598-020-74099-5

**Published:** 2020-10-12

**Authors:** Teruhisa S. Komatsu, Noriaki Okimoto, Yohei M. Koyama, Yoshinori Hirano, Gentaro Morimoto, Yousuke Ohno, Makoto Taiji

**Affiliations:** 1 Laboratory for Computational Molecular Design, RIKEN Center for Biosystems Dynamics Research (BDR), 6-2-3 Furuedai, Suita, Osaka 565-0874 Japan; 2 Drug Discovery Molecular Simulation Platform Unit, RIKEN Center for Biosystems Dynamics Research (BDR), 6-2-3 Furuedai, Suita, Osaka 565-0874 Japan

**Keywords:** Biophysics, Computational biology and bioinformatics, Drug discovery

## Abstract

We performed molecular dynamics simulation of the dimeric SARS-CoV-2 (severe acute respiratory syndrome corona virus 2) main protease (M^pro^) to examine the binding dynamics of small molecular ligands. Seven HIV inhibitors, darunavir, indinavir, lopinavir, nelfinavir, ritonavir, saquinavir, and tipranavir, were used as the potential lead drugs to investigate access to the drug binding sites in M^pro^. The frequently accessed sites on M^pro^ were classified based on contacts between the ligands and the protein, and the differences in site distributions of the encounter complex were observed among the ligands. All seven ligands showed binding to the active site at least twice in 28 simulations of 200 ns each. We further investigated the variations in the complex structure of the active site with the ligands, using microsecond order simulations. Results revealed a wide variation in the shapes of the binding sites and binding poses of the ligands. Additionally, the C-terminal region of the other chain often interacted with the ligands and the active site. Collectively, these findings indicate the importance of dynamic sampling of protein–ligand complexes and suggest the possibilities of further drug optimisations.

## Introduction

The pandemic of the new corona virus disease, COVID-19, is an urgent global issue. Currently, many research groups are trying to find effective medicines by repurposing approved drugs, using clinical, experimental, and computational approaches^1,2^. However, till date, no therapeutic agent has been approved to be effective against COVID-19 (except remdesivir in Japan). Here, we report the drug binding process of the severe acute respiratory syndrome coronavirus 2 (SARS-CoV-2) main protease (M^pro^, 3CL hydrolase), using large-scale molecular dynamics (MD) simulations. The M^pro^ protein is essential for processing the precursor polyprotein for replication of the virus. Owing to its crucial role, M^pro^ is one of the major targets for development of anti-SARS-CoV-2 drugs. The first X-ray crystal structure of M^pro^ was released on February 5, 2020^3^. Since then, the number of experimental structures has increased rapidly. These crystal structures enclose the structures of holo-M^pro^ (inhibitor covalently bound to M^pro^^3–7^ and inhibitor non-covalently bound to M^pro^) and ligand-free M^pro^^4,8^ are contained. Recently, Kneller et al.^8^ identified the room temperature X-ray structure of the ligand-free M^pro^ and compared it with the low temperature one of ligand-free M^pro^^4^ and N3 inhibitor covalently bound to M^pro^^3^. They found that the active site of M^pro^ had flexible conformation and the conformational change was induced by ligand binding. For drug repurposing for SARS-CoV-2 M^pro^, protease inhibitors of human immunodeficiency virus (HIV) are expected to be effective since HIV protease shows similar function as SARS-CoV-2. Many HIV protease inhibitors have been developed and clinical trials of the repurposed HIV protease inhibitors for COVID-19 are currently ongoing (e.g. ChiCTR2000029603). Among these inhibitors, China’s National Health Commission has recommended the use of HIV-1 protease inhibitors, lopinavir and ritonavir, as an ad hoc treatment for pneumonia caused by SARS-CoV-2. However, the results from an urgent randomised clinical trial, evaluating the efficacy of lopinavir–ritonavir in patients with COVID-19 in Wuhan, China, showed that no benefit was observed with lopinavir–ritonavir treatment beyond standard care for hospitalised adult patients^9^. Another HIV protease inhibitor, nelfinavir, is also one of the drug candidates against COVID-19. Nelfinavir showed suppression of growth of SARS-CoV in a cell-based experiment^10^. Although the mechanisms that underlie the inhibitory action of nelfinavir on SARS-CoV remain to be identified, the high sequence similarity (about 96%) between the M^pro^ of SARS-CoV-2 and that of SARS-CoV^11^ led us to hypothesise that nelfinavir may have promising activity against SARS-CoV-2 M^pro^. Furthermore, it has been recently reported that nelfinavir had anti-SARS-CoV-2 activity, as demonstrated in a cell-based experimental assay^12,13^. In addition, other HIV protease inhibitors such as indinavir, darunavir, and saquinavir, have been proposed as drug candidates against SARS-CoV-2 M^pro^, using computational studies^14–18^. These HIV protease inhibitors are repurposed drug candidates, some of which are already being tested in clinical trials. Recently it has been reported that the M^pro^ enzymatic activity could not be reduced by ritonavir, lopinavir, darunavir, and nelfinavir^6^. However, their efficacies against SARS-CoV-2 M^pro^ are yet to be fully confirmed.

In this study, we aimed to investigate the dynamics of the binding process of various HIV protease inhibitors to SARS-CoV-2 M^pro^. We performed all-atom MD simulations of the systems with the dimeric M^pro^ and seven HIV protease inhibitors (darunavir, indinavir, lopinavir, nelfinavir, ritonavir, saquinavir, and tipranavir), solvated in saline solution. Large-scale simulations, starting from ligand unbound states (28 simulations of 200 ns for each ligand), have been done using the massively parallel supercomputer. The results enabled a systematic investigation of the ligand access on the protein surface of M^pro^, and the frequently accessed sites on M^pro^ were classified, based on the contact between the ligand and the protein. These potential drug binding sites could be useful for further drug development/repositioning. Furthermore, we performed microsecond-scale simulations for the 23 protein–ligand complexes using the special-purpose computer MDGRAPE-4A^19^, which is designed for long-term MD simulations, in addition to using conventional supercomputers. Results revealed that the active site has a high flexibility and allows various binding poses of these ligands.

## Results

### Identification of ligand binding sites

**Figure 1:**
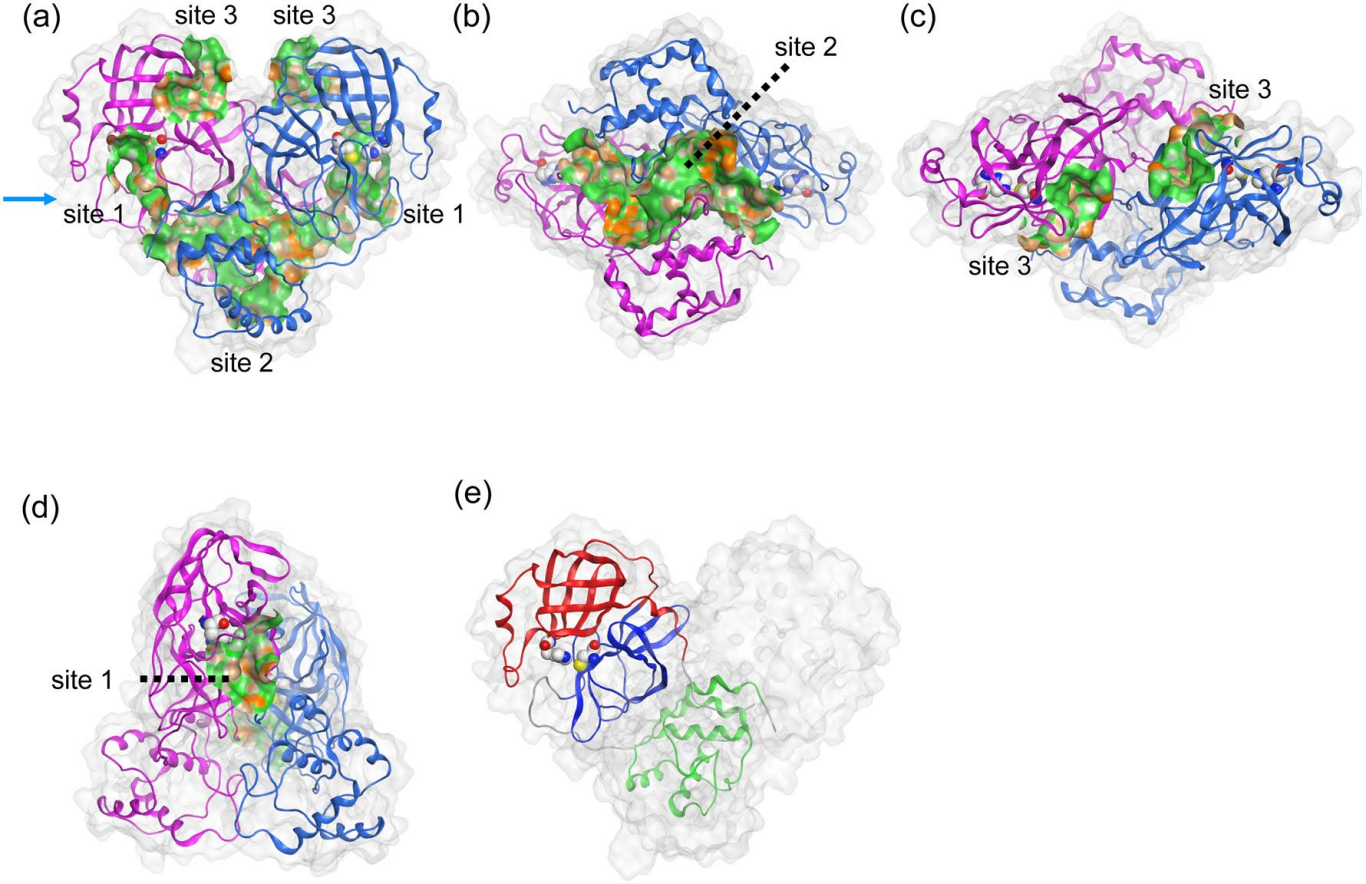
Structural information of dimeric M^pro^. The respective protomers of dimeric M^pro^ are shown in magenta and blue cartoon representations. The catalytic dyad, His41 and Cys145, are represented by the space-filling model. The three possible ligand binding sites were predicted. (**a**) Side view, (**b**) bottom view, (**c**) top view, (**d**) the view from the arrow of (**a**), and (**e**) the three domains of protomer (monomer) structure. Each protomer was composed of three domains: domains I, II, and III are residues 8–101 (red), 102–184 (blue), and 201–303 (green), respectively^3^. The lipophilic and hydrophilic regions in each site are depicted with green and orange.

First, the identification of the potential sites for drug binding was performed on the X-ray crystal structure^3^ of dimeric M^pro^, using the site finder module of Molecular Operating Environment (MOE)^20^. We found three representative drug binding sites on M^pro^ and named them as “sites 1–3” (Fig. 1). Site 1 was the orthosteric active site that had the catalytic residues, His41 and Cys145. Site 2, which was the largest site, was located near the interface of two domain III, and Site 3 was located at domain I.

Next, we investigated the access of drugs to these binding sites, in addition to other sites, on the fluctuating surface of M^pro^ by observing the dynamical trajectories obtained by direct MD simulations of dimeric M^pro^ with seven HIV inhibitors, darunavir, indinavir, lopinavir, nelfinavir, ritonavir, saquinavir, and tipranavir (Supplementary Fig. A1). When a drug accesses the M^pro^ surface, it is likely to form an encounter complex that is not tightly bound to the protein. We hypothesised that the stability and frequency of formation of the encounter complex would reflect the likelihood of the binding process between the drug and the protein. Hence, we thoroughly investigated the formation of the encounter complex by 28 simulations of 200 ns for each ligand (Supplementary Fig. B1).

**Figure 2:**
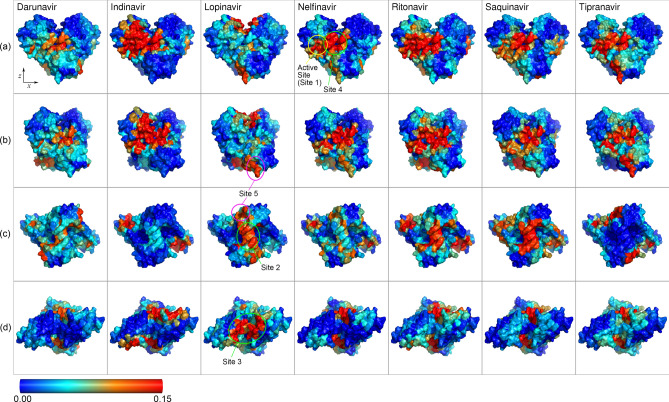
Heat maps of the contact frequencies for the seven ligands. Row (**a**): front view, (**b**): side view (rotate 30° around z axis), (**c**): bottom view (from -z axis), (**d**): top view (from + z axis). Frequent contacts at the active site were observed in indinavir, nelfinavir, ritonavir, and tipranavir. The major contacts at site 2 were lopinavir, ritonavir, and saquinavir, while site 3 was frequently visited only by lopinavir.

To analyse the formation ratio of the encounter complex, we calculated the contact map of each ligand to the protein, as shown in Fig. 2. These were calculated using last 100 ns (500 time points at every 0.2 ns) of each 200 ns simulation, and the threshold of a contact pair was set to 0.35 nm. The contact events on both chains of the dimer were accumulated, and a contact frequency was calculated as the number of events divided by the total number of samples. The results showed that most of the contacts were located at the predicted binding sites, as shown in Fig. 1. The frequent contacts with the active site (site 1) were observed for indinavir, nelfinavir, ritonavir, and tipranavir (Fig. 2a,b). Adjacent to the active site, a frequently visited site existed at the border of the chains, indicated as “site 4” in Fig. 2. It was a new site that was not considered as a major binding site in Fig. 1. Frequent visits to site 4 were observed for all ligands, except lopinavir. The contact frequency to site 2 (Fig. 2c) was high for lopinavir, ritonavir and saquinavir, modest for darunavir, indinavir and nelfinavir, and weak for tipranavir. In domain III (Fig. 1e), another shallow site “site 5” was observed for lopinavir, nelfinavir, and tipranavir (Fig. 2b,c). The contact frequency to site 3 was generally low, except for lopinavir (Fig. 2d). These absolute values of contact strength should be interpreted carefully since we could observe only a few unbinding events and they did not accurately reflect the quantified values in equilibrium.

To classify the frequently accessed sites, we performed clustering analysis of the contact maps for whole trajectories of 200 ns (1000 time points at every 0.2 ns). First, we performed hierarchical clustering analysis and chose nine clusters classification. Next, we performed *k*-means clustering analysis with *k* = 9, using the results of the hierarchical clustering analysis as the initial cluster centres for *k*-means clustering (Supplementary information C and D). Based on the obtained classification of the sites, the number of transition events in the trajectories and the binding free energies (estimated by the molecular mechanics generalized-Born surface area (MM-GB/SA) method^21,22^) are summarised in Table 1. The time course of transitions among the classified sites and the binding free energies for each trajectory of the seven ligands are shown in Supplementary Figs. E1–7. As shown in Fig. 2, site 3 was only frequently visited by lopinavir. Site 5 experienced a comparable number of on and off events, which was attributed to its relative shallowness compared to the other sites. The active site (site 1), site 2, and site 4 were rather stable for most of the ligands. Among the seven ligands, indinavir, nelfinavir, ritonavir, and tipranavir had similar profiles, with rather high counts of contact events to the active site and site 4. Ritonavir also bound to site 2 frequently, and a few unbinding events from the active site were observed. In addition, darunavir, lopinavir, ritonavir, and saquinavir had visited the active site, but the number of events and the free energies were competitive with the other sites: site 2, 3 (lopinavir only), and 4. Since it is difficult to compare the binding energies of the different ligands by MM-GB/SA energies, the selection of the best candidate was not possible. However, by comparing Δ*G* of the active site with respect to the other sites for each ligand (Table 1), indinavir, nelfinavir, and tipranavir could be considered as possible candidates for further drug optimisation.

**Table 1:** The number of events and the binding free energies estimated by the MM-GB/SA method in 28 trajectories of 200 ns for each ligand.

	Active site	Site 2	Site 3	Site 4	Site 5	Others
**Darunavir**						
On/off	7/5	***4/0***	*1/0*	5/2	8/7	14[9]/25
∆*G* ave	**− 28.4 **(4.2)	**− **24.2 (4.2)	**− **17.2 (2.0)	**− **26.6 (6.9)	**− **18.8 (3.6)	**− **22.3 (5.2)
∆*G* min	**− **32.7	**− **32.6	**− **17.9	**− 38.6**	**− **21.2	**− **30.3
**Indinavir**						
On/off	**13/4**	*2/0*	6/6	10/5	2/2	15[5]/31
∆*G* ave	− 33.2 (7.5)	− **33.7 **(6.6)	− 22.0 (5.7)	− 28.4 (5.9)	− 24.6* (6.6)	− 26.5 (5.8)
∆*G* min	− **56.1**	− 42.8	− 28.8	− 43.5	− 26.5	− 41.1
**Lopinavir**						
On/off	*4/1*	8/4	**9/3**	3/2	7/3	12[8]/30
∆*G* ave	− 23.0 (4.0)	− **29.4 **(6.2)	− 28.5 (5.3)	− 20.8 (5.3)	− 25.6 (5.8)	− 34.5 (6.6)
∆*G* min	− 32.7	− **39.1**	− 34.6	− 31.3	− 30.4	− 50
**Nelfinavir**						
On/off	6/1	4/1	1/1	***8/1***	4/1	3[9]/21
∆*G* ave	**− 31.2 **(8.9)	**− **24.2 (3.6)	–	**− **26.6 (5.8)	**− **33.3 (3.9)	**− **25.6 (4.4)
∆*G* min	**− 51.5**	**− **30.3	–	**− **40.6	**− **37.6	**− **36.7
**Ritonavir**						
On/off	**9/3**	*5/0*	3/2	**10/4**	7/5	14[5]/34
∆*G* ave	− 40.0 (6.4)	− 38.2 (7.9)	− 27.6* (2.9)	− **45.3 **(11.0)	–	− 42.1 (4.5)
∆*G* min	− 51.5	− 53.6	− 25.8	− **63.9**	–	− 50.5
**Saquinavir**						
On/off	6/2	**9/3**	3/3	***6/0***	3/1	8[8]/26
∆*G* ave	− 32.6 (4.8)	− **35.3 **(9.8)	− 25.8* (4.3)	− 32.3 (6.8)	–	− 20.8 (3.3)
∆*G* min	− 39.1	− **47.8**	− 30.3	− 44.4	–	− 29.4
**Tipranavir**						
On/off	***10/3***	0/0	3/2	7/3	7/3	11[8]/27
∆*G* ave	**− 25.9 **(7.6)	–	**− **21.4 (3.1)	**− **23.8 (6.6)	**− **24.0 (7.5)	**− **24.4 (5.3)
∆*G* min	**− 42.6**	–	**− **34.2	**− **36.9	**− **34.1	− 36.9

The classification was based on the clustering analysis described in Supplementary information C. The events were counted after smoothing by taking majority for 71 data points, sampled every 0.2 ns. The number in square brackets in the others column indicates the number of trajectories that stayed on it during whole simulation period. The sites printed in bold type had the largest fraction at final states (the largest number of “on” events minus “off” events), except for the others category, the sites printed in italic type had the smallest ratio of off/on events, and the sites printed in bold and italic type satisfied both the conditions. The rows “∆*G* avg” and “∆*G* min” indicate the average (with the standard deviation in parenthesis) and minimum binding free energy in a unit of kcal/mol, respectively. The energies were omitted when the solvent-accessible surface area (SASA) of each ligand was above 50% of the SASA of the solvated ligand (Supplementary Information E). The lowest energies among sites 1–5 are printed in bold type. The average energies were calculated from the last 100 ns, except for values marked by asterisk, which were taken from the full trajectories. The minimum energies were taken from the averaged energies over 15 continuous points, every 0.2 ns of the whole 200 ns trajectory.

**Figure 3:**
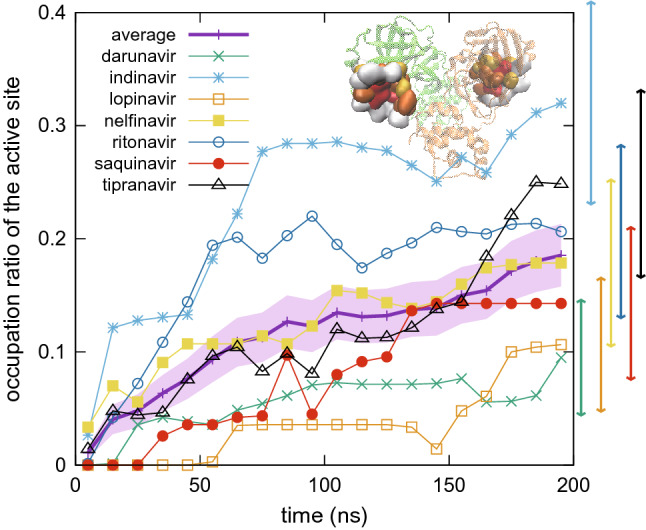
Time course of occupation ratio at the active site. The figure shows mean occupation ratio averaged in a time span of every 10 ns over 28 trajectories for each ligand. The average over seven ligands is also shown with thicker line (standard error with shaded area). Standard error for final 10 ns of each ligand is shown at the right side of the graph (see Supplementary F for the estimation of errors). Inset shows the active site residues (see Supplementary Fig. D1). Occupation ratios for the other sites are shown in Supplementary Fig. F1.

Figure 3 shows the time course of the occupation ratio in the active site. The ratio averaged over the seven ligands increased with time compared to the other sites shown in Supplementary Fig. F1. This suggested that the active site in fact could be a potential drug binding site. It required a minimum simulation duration of 100 ns to observe these tendencies, for e.g. tipranavir reached the active site after 100 ns. After reaching the active site, the ligands still exhibited equilibration dynamics, which should be investigated with a further longer simulation. Although the number of samples for each ligand in this study did not allow a detailed comparison of the binding affinities to the active site among the ligands, we anticipated that the tendency of binding (Fig. 3) reflects the likelihood to form encounter complexes at the active site, i.e. tipranavir and indinavir were more likely to bind to the active site of M^pro^ compared to lopinavir and darunavir. Additionally, as a negative control experiment, similar MD simulation was performed using lamivudine triphosphate, which is also an approved drug but has different molecular structure from that of HIV drugs. It clearly exhibited infrequent access to the active site (Supplementary information G).

While MD-based free energy calculation is necessary to provide precise selections of drug candidates, it requires proper complex structures. We extended the duration of simulations up to a microsecond (µs) for 23 trajectories, arbitrarily selected from the trajectories in which the ligands attached to the active site at 200 ns. In 20 of 23 trajectories, the ligands remained bound to the active site during 1 µs. For these 20 trajectories of 1 µs long, the binding free energies were estimated by MM-GB/SA method (Supplementary information H). By comparing Table 1 and Supplementary Table H1, the minimum and average binding free energies decreased for all ligands (except for the slight increases in the minimum energy of nelfinavir and in the average one of saquinavir). This observation indicated that the current simulation time of 200 ns was insufficient for the equilibrium analysis. In the next section, we analysed the active site structures and ligand binding poses to understand the key factors involved in binding and their dynamical properties.

### Conformational variations upon ligand binding

**Figure 4:**
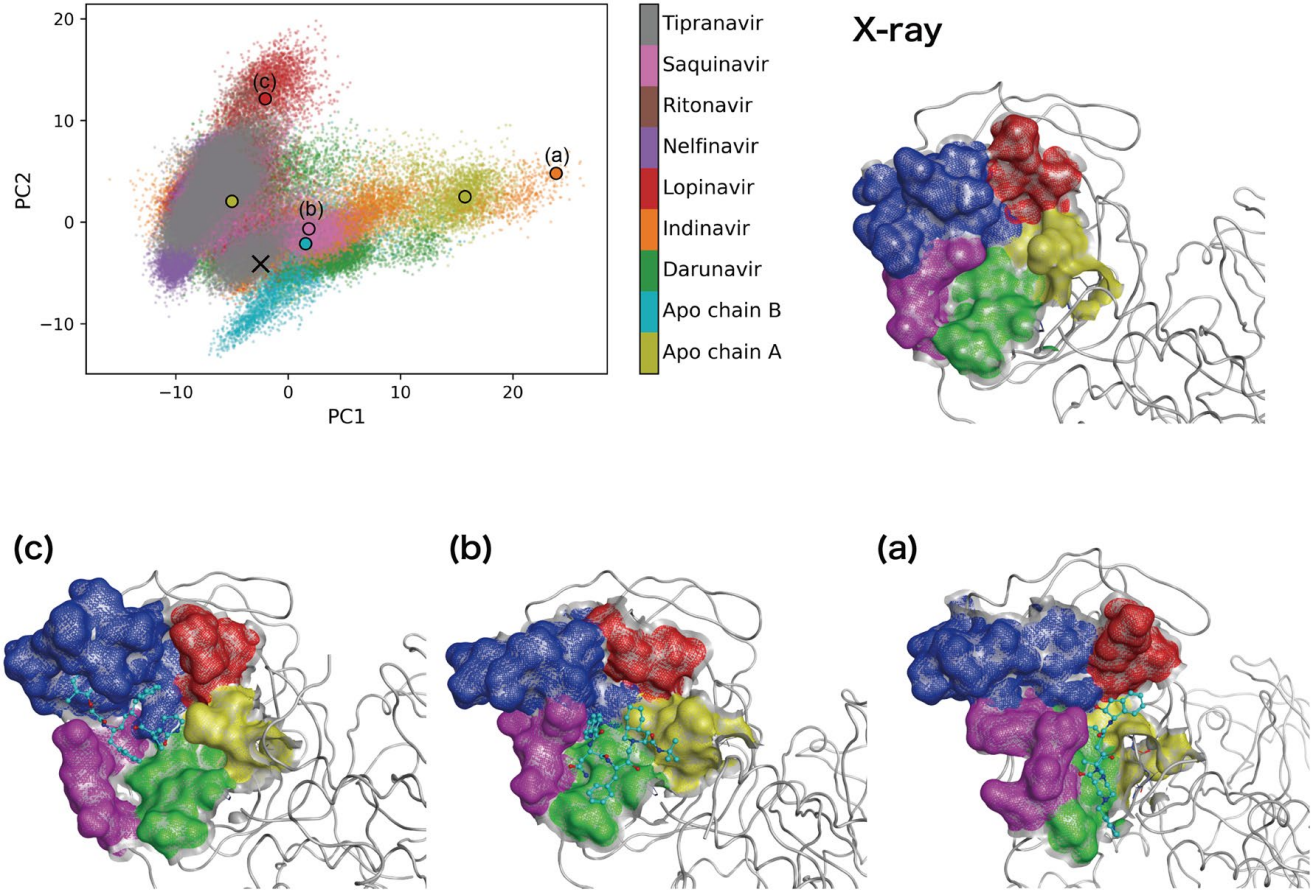
Conformational diversity of the M^pro^ active site. The projection of the first two principal components (PC1 and PC2) is shown. The contribution ratios of first two principal components (PC1 and PC2) were 36.1% and 12.9%, respectively. The conformations of the active site, corresponding to the cross mark and (**a**)–(**c**) in the projection, are shown: the cross mark and (**a**)–(**c**) are the crystal structure (PDB ID: 6LU7) and simulation structures of indinavir-bound M^pro^, saquinavir-bound M^pro^, and lopinavir-bound M^pro^ systems, respectively. The five regions of the active site, (1) residues 24–27, (2) 41–54, (3) 140–145, (4) 163–168 and 172, and (5) 187–192, are coloured in red, blue, yellow, green, and magenta, respectively. The representative conformations for the active site of apo-M^pro^ system are shown in Supplementary Fig. J3. The Apo chain A and Apo chain B correspond to the trajectories of the active sites of the chain A and the chain B of the apo-M^pro^ system, respectively.

To explore the conformational variations in the M^pro^ active site, a principal component analysis of Cα atoms of 37 amino acid residues (residue 24–27, 41–54, 140–145, 163–168, 172, and 187–192) contained in the active site (see Supplementary information J) was performed for 20 ligand-bound MD trajectories together with another MD trajectory of M^pro^ without ligand (apo-M^pro^). The projection of the first two principal components (PC1 and PC2) characterised the conformational diversity of the active site (Fig. 4). In the apo-M^pro^ system, while the MD trajectory of the active site of A-chain widely distributed along the PC1, that of B-chain distributed near the crystal structure. In contrast, the MD trajectories of the active sites of the ligand-bound systems distributed wider along PC1 and PC2, as compared with those of apo-M^pro^ system. Especially, since the second eigenvector was related to the opening motion of the two loop regions (residue 41–54 and 187–192; Supplementary Fig. J2), it was considered that the conformations of the active site with large PC2 value (> 10) were induced particularly by the interactions of the ligands (see Fig. 4c and Supplementary Fig. J2). Furthermore, the conformations of the active site (obtained from the MD simulations) largely changed from that of the crystal structure, and various conformations emerged in the ligand-bound systems as well as apo-M^pro^ system. Although it was difficult to systematically classify the active site conformations after the binding of the different ligands, its characteristic conformations could be visualised (examples shown in Fig. 4a,c, lopinavir-bound M^pro^ and indinavir-bound M^pro^ systems).

**Figure 5:**
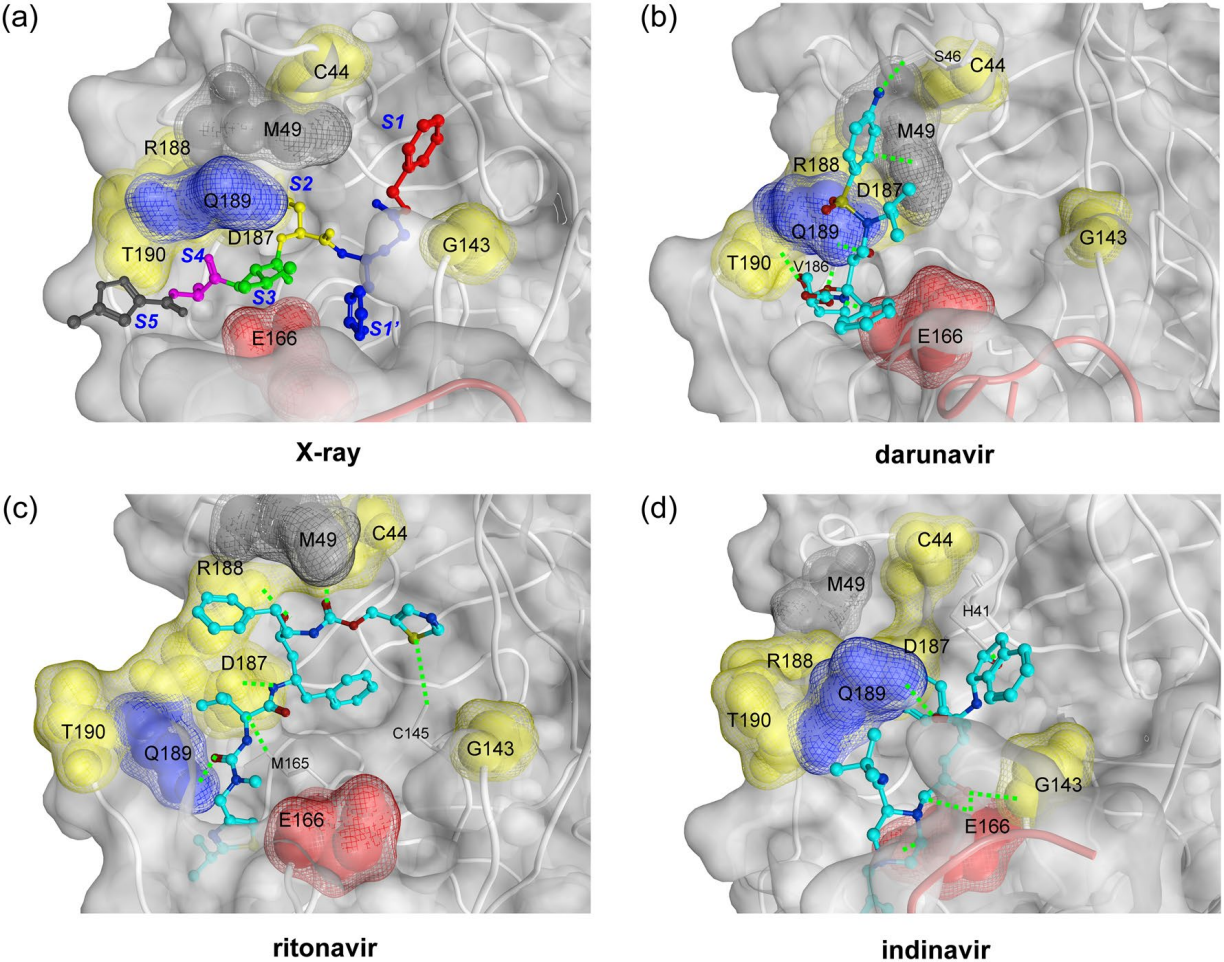
Key active site residues for ligand binding. (**a**) The key amino acids were determined with the representative appearance rate of the interaction fingerprints (RAIF) and are highlighted with space-filling models in red (Glu166), blue (Gln189), yellow (Cys44, Gly143, Asp187, Arg188, and Thr190), and grey (Met49). First two residues, Glu166 and Gln189, were commonly utilised for multiple ligands, while others for single ligand (see main text and Supplementary Table K1). This figure is depicted on the crystal structure of M^pro^-N3 inhibitor complex (PDB ID: 6LU7). The P1′, P1, P2, P3, P4, and P5 residues of N3 inhibitor is shown with ball and stick models in red (P1′), blue (P1), yellow (P2), green (P3), magenta (P4), and grey (P5), and the S1′, S1, S2, S3, S4, and S5 indicate the subsites corresponding to P1′–P5 sites of N3 inhibitor^3^. (**b**) The representative binding pose of darunavir-bound M^pro^ system is shown. The ligand interacted with Ser46, Met49, Glu166, Val186, Gln189, and Thr190. (**c**) The representative binding pose of ritonavir bound M^pro^ system is shown. The ligand interacted with Cys44, Cys145, Met165, Asp187, Arg188, and Gln189. (**d**) The representative binding pose of indinavir-bound M^pro^ system is shown. The ligand interacted with His41, Gly143, Glu166, and Gln189. In (**b**)–(**d**) structures, the detected hydrogen bonds (Supplementary information K) are shown with green dotted lines. The ligands are shown with ball and stick models.

Next, we investigated how each ligand interacted with the active site residues in the MD simulations. To detect the important protein–ligand interactions, we analysed the interaction fingerprint which could enable the identification of the existence of the ionic and the hydrogen bonds between the protein and the ligand for each snapshot in MD trajectories. Observing the representative appearance rate of the interaction fingerprints (RAIF) in Supplementary Table K1, the seven key active site residues were found to have comparatively large contribution (residues 44, 143, 166, 187–190 with RAIF > 20%) and were speculated to play an important role in the ligand binding. In addition, by performing clustering analysis of the fingerprints, we picked three representative binding poses for each ligand from top three classified clusters. Figure 5 shows the typical binding poses with the seven key residues, as highlighted (Supplementary Figs. K1–7 show full set of binding poses). Based on the observation of the binding poses, we found that the various binding poses were contained in MD simulations of each ligand-bound M^pro^ system, and the variety of binding poses resulted from not only the initial conformation of the encounter complex but also from the conformational refinement and/or equilibrium dynamics within each 1 μs MD simulation. In addition, it was clear that the conformations of the active site (obtained from MD simulations) were markedly different from that of the crystal structure that was covalently bound with the inhibitors, with respect to the positions of the key residues. This suggested that the shapes of the subsites observed in the crystal structure^3^ changed significantly in MD simulations (see Fig. 5a).

The two key residues, Glu166 and Gln189, were especially noted because they commonly formed effective interactions (RAIF > 20%) with multiple ligands. Glu166 formed effective interactions with five ligands (darunavir, indinavir, lopinavir, nelfinavir, and tipranavir) (Supplementary Table K1). The interaction was influenced mainly by conformational change of Glu166 side chain. Gln189 also formed effective interactions with three ligands (darunavir, ritonavir, and saquinavir), and had comparable interactions with indinavir (RAIF = 19.1%) and nelfinavir (RAIF = 18.8%). Thus, Gln189 could be utilised as a key residue for a broad range of ligands. Gln189, which is known to be quite flexible in the M^pro^ of SARS-CoV^23^, belongs to the loop region (residue 187–192) that is closely related to the PC1-2 eigenvectors (Supplementary Fig. J2). Gln189 could maintain effective interactions with a variety of ligands by utilising the flexibilities in its side chain and the backbone of the loop. Each of the other five key residues formed effective interactions with only one of the seven ligands. The three residues: Asp187, Arg188, and Thr190 (present in the same loop as Gln189) might have relatively less chance to form effective interactions because of their location within the loop and the preferred orientation of their side chains. The remaining two residues: Cys44 and Gly143, formed effective interactions with darunavir (Supplementary Table K1), which suggested potential utilisation of these key residues for a specific class of drugs. Besides the seven key active site residues noted above, Met49 in the loop region (residue 41–54) formed moderate interactions (RAIF of 5.0–16.5%) with the seven ligands. As Met49 is known to play an important role in accommodating a substrate peptide for the M^pro^ of SARS-CoV^24^, it might largely contribute to the molecular recognition for drug development against M^pro^. In addition to these commonly utilised interactions, we also found some of the interactions formed uniquely to a ligand (Supplementary Fig. K8) which implied a broad possibility of conformational variation in the active site specifically induced by the ligands. These results indicated that there were diverse interaction patterns of the respective ligands in the flexible active site of M^pro^.

## Discussion

We investigated the access to the drug binding sites in SARS-CoV-2 M^pro^, using seven HIV inhibitors as potential lead drugs. The frequently accessed sites on the M^pro^ were classified based on the contact between the ligand and the protein. Although the limited length of the simulations may give statistics only for encounter complexes, it can provide a list of potential drug binding sites which can be employed for further drug development/repositioning. The microsecond-scale simulations of the active site complexes of M^pro^ and ligands revealed a wide variation in the shapes of the active site and also in the binding poses of the ligands. This suggested that the surface of the M^pro^ is rather flexible, and conformational change due to induced fit between the ligand and the protein was a dominant factor affecting the binding processes. Thus, MD simulation could be an effective tool to investigate the ligand binding on the current M^pro^ system.

Molecular docking is a practical computational method for drug discovery. The conventional molecular-docking approach involves rigid receptor-flexible ligand docking and uses a single protein structure, such as the X-ray crystallographic structure. However, it could be difficult to adopt this approach for a target like the M^pro^ active site, which has high flexibility. Here, we performed preliminary conventional molecular dockings using the representative MD simulation structures as well as the crystal structure and the drug-like compound library (Supplementary information L). Supplementary Table L1 showed that the distinctive set of promising inhibitor candidates was obtained by molecular dockings based on each employed protein structure. This suggested that the molecular docking using a single protein structure was not sufficient for drug discovery. Hence, the protein structural information obtained from our MD simulations could be utilised for structure-based drug design strategies, including the ensemble docking. For a detailed comparison of the binding affinity among ligands, an estimation of the precise protein–ligand complex structure is crucial and can be achieved by MD simulations with longer duration. Recently, several studies on promising inhibitors of M^pro^ system, using molecular docking and MD simulations, have been reported^14–18,25,26^. These studies proposed several HIV-1 protease inhibitors as promising inhibitor candidates of M^pro^. The results varied depending on the computational conditions, such as the used M^pro^ structure and the docking program. In particular, the influence of molecular docking on the M^pro^ structure was also supported by the results of our preliminary molecular dockings (Supplementary information L). However, since our current work mainly focused on the analysis of the dynamical access of drug molecules to the surface of the M^pro^, a direct comparison of our current work with these studies is not feasible. Moreover, our results suggested that the non-specific binding to sites other than the active site should be considered while designing drugs.

**Figure 6:**
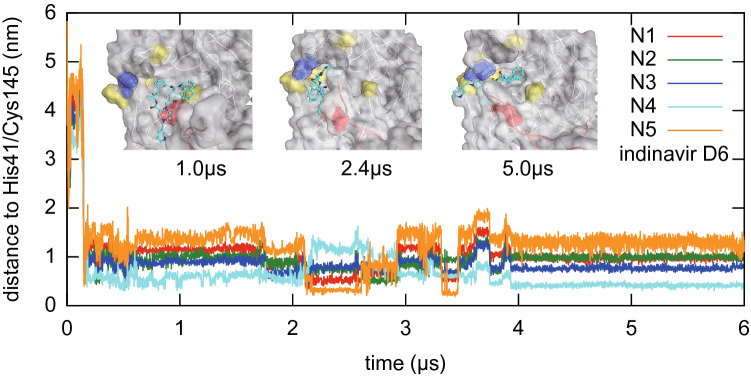
Ligand flipping during 6 μs MD simulations. Minimum distances between a nitrogen atom in the ligand (indinavir) and the catalytic residues His41/Cys145 are plotted. These distances reflect binding pose of the ligand, and thus flipping events are visualised as a sudden switching of these distances. Inset shows ligand poses at 1.0, 2.4, and 5.0 μs, where surfaces of the active site are coloured as in Fig. 5.

To explore longer temporal behaviour of ligand binding at the active site of the M^pro^, three pilot simulations for indinavir, nelfinavir, and tipranavir were performed by extending three trajectories to reach 6 µs (Fig. 6 and Supplementary Fig. M1). It was observed that the ligands stayed at the active site for μs scale. However, it was also observed that the ligands exhibited flipping of the binding pose a few times in 6 µs. These observations suggest that these binding states might be rather loose, and such loose binding states may be a typical or essential class of the small-molecule ligand binding state to the M^pro^ active site. There is a possibility to develop a more effective drug that can bind tightly to the M^pro^ active site with high enthalpy difference. We hope systematic investigation of longer temporal behaviour in future researches will give us a perspective view of the drug binding dynamics.

**Figure 7:**
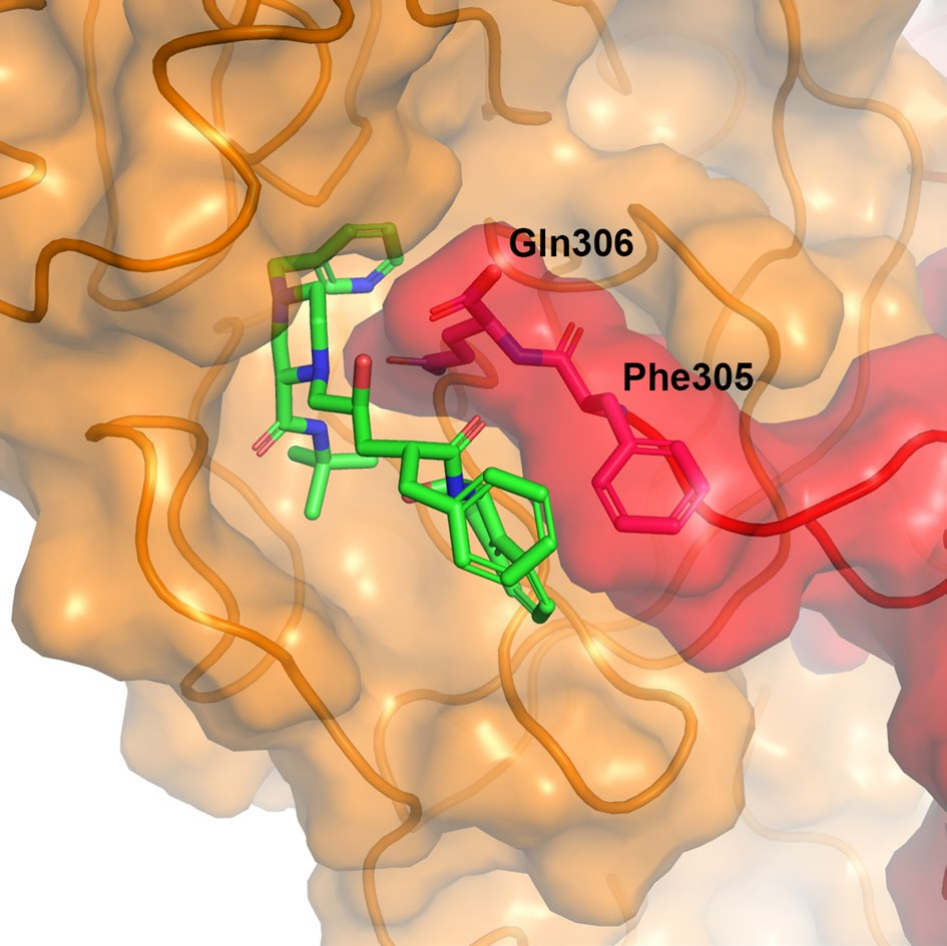
C-terminal region interacting with indinavir at the active site. Similar interactions were observed for the other ligands. The chain in orange corresponds to the chain that has the active site occupied by indinavir, and the one in red (C-terminal) corresponds to another chain of the dimer. This snapshot was taken at 2.4 µs from the trajectory shown in Fig. 6.

Another noteworthy finding was the interaction between the ligands and the C-terminal residues of the other chain of the dimer, observed for all ligands. Figure 7 shows the typical snapshot of such interactions in the case of indinavir. In the extended simulations of 1 µs, the C-terminal residue, Gln306, stayed within a minimum distance of 0.35 nm to the ligand for 8% of the time duration (from 200 to 1000 ns, averaged over 20 simulations) (Supplementary Table N1). In addition, in several cases, the region entered the active site (Supplementary Fig. N1). Similar observations were noted in the clustering analysis of protein–ligand contacts and interactions. Since M^pro^ is known to autoprocess the N- and C-terminals of the precursor protein of itself^27,28^, it is reasonable to observe the interaction of the C-terminal region and the active site. This fact also suggested that the observed C-terminal interaction might stabilise the substrate or drug interactions. The observed dynamic interaction, revealed by the MD simulations, would be another important factor to be considered in drug design.

The analysis on ligand access to the surface of the M^pro^ provided several drug binding sites, in addition to the orthosteric active site. Among these sites, sites 2 or 3, (located at the interface of the dimeric M^pro^) were relative frequently accessed by lopinavir, ritonavir, and saquinavir and may be worth exploring. While the ligand binding to these two sites may not directly inhibit the enzymatic reaction, there is a possibility to influence the dimerisation and structural stabilisation of the dimeric M^pro^. It would be interesting to elucidate the roles of these sites by simulating the binding of the other drug molecules and the substrate peptides. Further, a study on the detailed mechanism to recognise the specific amino acid sequences in the active site is an interesting target of MD simulations in drug designing, including allosteric inhibitors. Our next goal is to propose an atomic level drug design strategy against the M^pro^ by integrating the dynamical information of various binding process.

## Methods

To simulate the binding process of seven HIV protease inhibitors (darunavir, indinavir, lopinavir, nelfinavir, ritonavir, saquinavir, and tipranavir) to the SARS-CoV-2 M^pro^, the initial structures were built based on the X-ray crystal structure of the holo SARS-CoV-2 M^pro^ (Protein Data Bank^29^ entry: 6LU7^3^). Since the crystal structure was the M^pro^ with the covalently bound inhibitor, the structure of the inhibitor-unbound M^pro^ (apo-M^pro^) was prepared by removing the covalently bound inhibitors. In addition, each HIV protease inhibitor was initially placed at least 1.5 nm apart from the active sites of SARS-CoV-2 M^pro^ (see Supplementary Fig. B1). The initial structure of the M^pro^ with an HIV inhibitor was then solvated in a cubic box of TIP3P water molecules. In addition, chlorine and sodium ions (0.154 M) were added to neutralise the system. The system of apo-M^pro^, without covalently bound ligand, was also prepared in a similar manner. Amber FF14SB^30^ was used for the M^pro^ protein, and general amber force field (GAFF)^31^ was used for seven HIV protease inhibitors. The partial charges for the ligands were calculated at the RHF/6-31G(d) level with Gaussian16^32^ and the restrained electrostatic potential method^33,34^. ParmEd^35^ was used to convert Amber topologies to GROMACS^36^ formats. VMD^37^ and PyMOL^38^ were used for visualisation.

All MD simulations were performed on the massively parallel supercomputer HOKUSAI Big Waterfall (BW) system at RIKEN ICS, or the special-purpose, specialised for faster calculation of MD, computer MDGRAPE-4A^19^ (the advanced version of MDGRAPE-4^39^) at RIKEN BDR. All hydrogen bonds and TIP3P waters were constrained with the methods summarised in Supplementary Table Y1. The periodic boundary conditions were applied to the system, and the long-range Coulomb interactions were treated with the method described in Supplementary information Y and Z, with a direct space cutoff distance of 1.3 nm. After the energy minimisation of each solvated system, the system was heated to 300 K for 1 ps with the integration time step of 0.5 fs, without constraints. Then, 100 ps MD simulations, with a time step of 2 fs, under NPT ensemble (*P* = 1 bar and *T* = 310 K), were performed to adjust the size of the simulation box. For each HIV inhibitor, the above relaxation protocol was applied for fourfold variation in the initial location of the inhibitor (Supplementary information B), and sevenfold variation produced by randomising velocities with different random seeds. As a result, totally 28 initial conditions were prepared for the 200 ns production runs on HOKUSAI-BW (with a time step of 2.5 fs) under NVT ensemble (*T* = 310 K). The trajectories of each system were saved for every 100 ps (2000 conformations in each MD trajectory), and most of the analysis were done on snapshots every 200 ps. In addition, 23 trajectories were picked for extended simulation of 1 µs duration (11 on MDGRAPE-4A and 12 on HOKUSAI-BW), and 3 trajectories in these trajectories were extended further to 6 µs duration on MDGRAPE-4A. The simulation of 1.8 µs trajectory for apo-M^pro^ and pilot runs in the early stage of this study were also performed on MDGRAPE-4A. Additional simulation to search negative control was partly performed on HOKUSAI Sailing Ship (SS) system.

## Supplementary information

Supplementary Information.

## Data Availability

Raw trajectory data analysed in this paper and movie examples are available at the zenodo repository^40^.
