# Supplementary material for: Drug binding dynamics of the dimeric SARS-CoV-2 main protease, determined by molecular dynamics simulation
Source: Sci Rep. 2020 Oct 12;10:16986. doi: 10.1038/s41598-020-74099-5 (PMC7550358; doi:10.1038/s41598-020-74099-5)
Supplement: Supplementary file 1 — Supplementary Information. [file 41598_2020_74099_MOESM1_ESM.pdf]

# Supplementary information

## Drug binding dynamics of the dimeric SARS-CoV-2 main protease, determined by molecular dynamics simulation

Teruhisa S. KOMATSU, Noriaki OKIMOTO, Yohei M. KOYAMA,
Yoshinori HIRANO, Gentaro MORIMOTO, Yousuke OHNO,
and Makoto TAIJI

Laboratory for Computational Molecular Design,
RIKEN Center for Biosystems Dynamics Research (BDR), JAPAN

**Contents:**

A. Seven HIV inhibitors used in this study.
B. Initial locations of ligands.
C. Clustering analysis of the contact data matrix.
D. Robustness of the clustering analysis.
E. Time series of contact positions of ligands and MM-GB/SA binding free energies.
F. Time course of occupation ratio in the classified contacts.
G. Negative control simulation infrequent binding to the active site.
H. MM-GB/SA free energy for 1 μs trajectories.
J. Principal component analysis for characterising the active site Conformations.
K. Representative binding poses for MD simulations of respective ligand-bound $M^{pro}$ systems.
L. Molecular docking using the X-ray crystal and MD simulation structures.
M. Binding pose flipping over long time scale of MD trajectories.
N. Interaction of the C-terminal residue of the other chain of the dimer with ligands and the drug binding site.
Y. Summary of numerical methods.
Z. Computation of Coulomb forces for MDGRAPE-4A.
References in Supplementary information

## Supplementary information A. Seven HIV inhibitors used in this study.

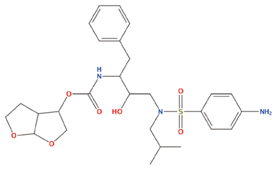

mw: 547.673

darunavir

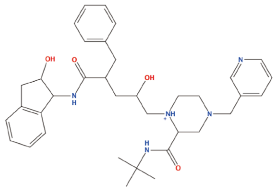

mw: 614.811

indinavir

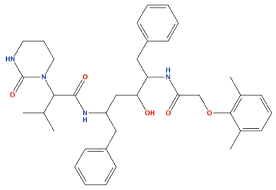

mw: 628.814

lopinavir

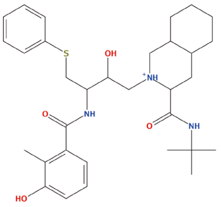

mw: 568.803

nelfinavir

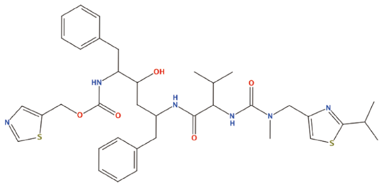

mw: 720.960

ritonavir

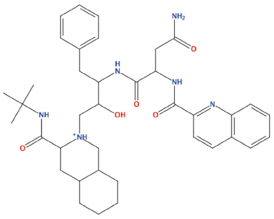

mw: 671.863

saquinavir

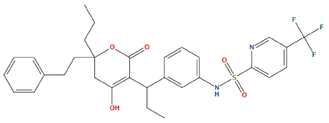

mw: 602.674

tipranavir

Supplementary Figure A1. Seven HIV inhibitors used in this study. Numerals indicate molecular weight.

## Supplementary information B. Initial locations of ligands. [TOP]

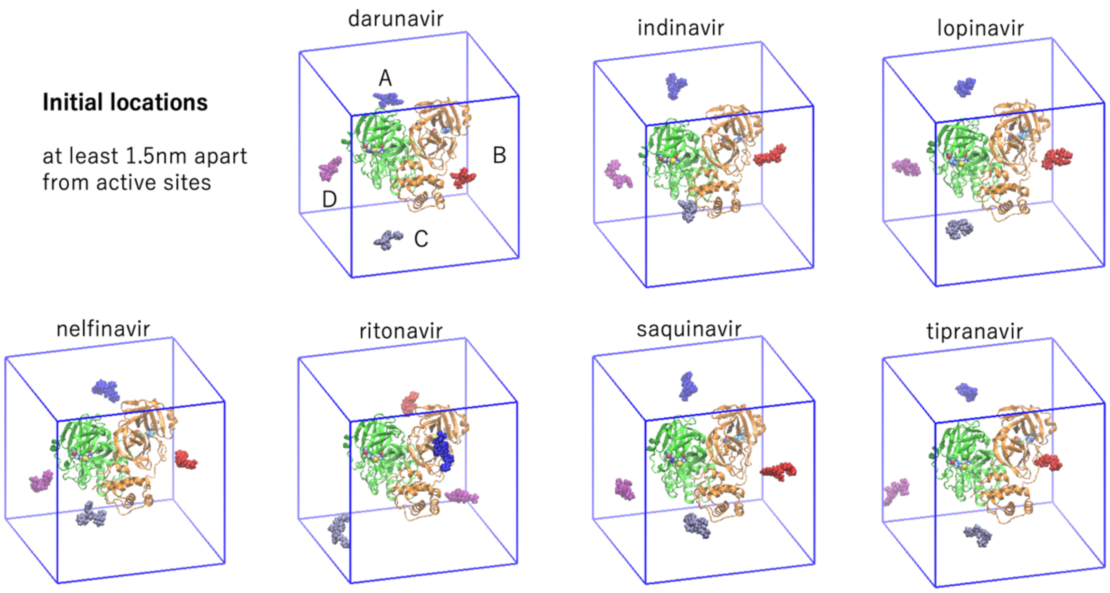


Supplementary Figure B1. Schematics for initial locations of ligands. We employed 4 variations of unbounded locations, A (blue), B (red), C (grey), and D (purple), arbitrary chosen at least 1.5 nm apart from the active sites of $M^{pro}$. For each initial location, seven variations of randomised initial velocities were employed for the production runs. In total 28 variations of production runs were performed for each ligand.

## Supplementary information C. Clustering analysis of the contact data matrix. [TOP]

### *Hierarchical clustering analysis of the contact data matrix:*

Minimum distances between amino acid residues of $M^{pro}$ dimer and each drug were computed by *gmx pairdist* command implemented in GROMACS[1]. The contact strength between a residue and the drug was defined as one if the minimum distance of the either residue in the dimer is less than the threshold length $d$ = 0.35 nm and zero otherwise. Here, the distances were measured among all the atoms of the residue and those of the drugs, including hydrogen atoms. Since $M^{pro}$ is a homodimer, we did not discriminate the contact from the either monomer. Then, the contact vector size $m$ becomes 306, the number of amino acid residues of the $M^{pro}$ monomer. To find the common contact patterns among seven drugs, we concatenated overall data points of seven drugs. The total data points $n$ is (7 drugs) × (4 initial drug positions) × (7 initial randomised velocities) × (200 ns / 200 ps) = 196,000. The hierarchical clustering analysis of the contact data matrix with Ward's method[2] was performed with *fastcluster* package[3] for Python, which only requires the memory usage on the order of $n \times m$. The dendrogram of the clustering analysis is shown in Fig. C1, where cluster identifiers (*clsid*) at level 9 (at which just top 9 clusters are classified) are shown on the green line.

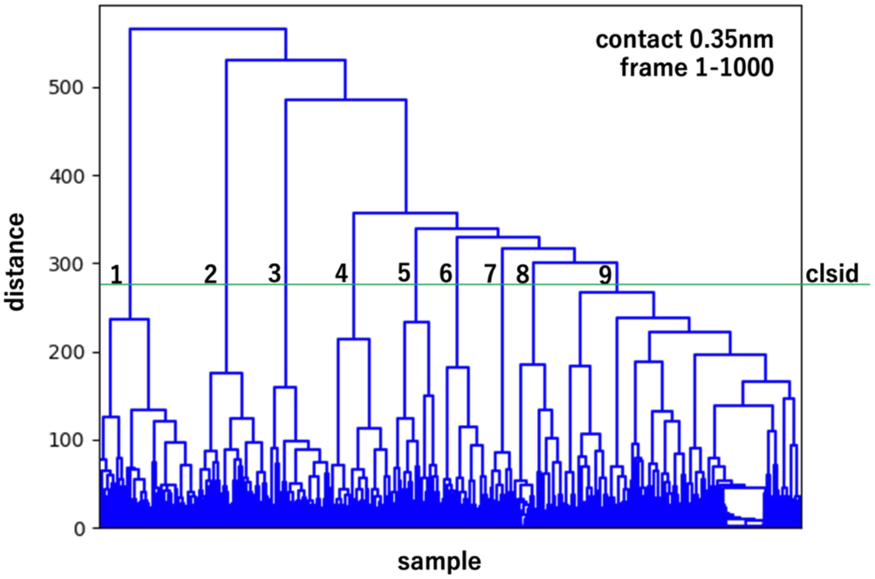


Supplementary Figure C1. Dendrogram for hierarchical clustering analysis. Top 9 clusters are shown with their cluster identifiers (clsid).

***K-means clustering analysis based on the hierarchical clustering result:***

Based on the result of the hierarchical clustering analysis described above, we have further refined the classification of the binding sites by using *k*-means clustering analysis with Hartigan-Wong algorithm[4] implemented in R[5]. As seen in Supplementary information D, the hierarchical clustering analysis at level=9 gives sufficiently robust classification of the binding sites. Based on this observation, the contact data matrix was reanalysed by using *k*-means clustering analysis of *k*=9 with the initial cluster centres defined by the hierarchical clustering analysis at level=9. As to the robustness of the *k*-means clustering result, we have confirmed that the same classification results obtained by using the initial set of centres defined by the hierarchical clustering analysis for more sparsely sampled (every 2ns) total 19600 data. Comparing the hierarchical clustering analysis and the *k*-means clustering analysis, the classification unchanged for 90% of data points as summarised in Table C1. We have visually inspected major difference between the result obtained by the hierarchical clustering and that by *k*-means clustering and observed that *k*-means clustering gives reasonable classification (especially trajectories of indinavir A3, D6, D7). Table C2 shows the list of the residues belong to each cluster, where residues of mean contact ratio greater than 0.2 are shown with bold. See below for the definition of the mean contact ratio.

***Identify cluster as a nearest cluster for a given contact vector:***

Above procedures (*k*-means clustering after Ward hierarchical clustering) give us *clsid* for each data point (snapshot). This *clsid* can be also obtained as a cluster id of minimum distance, because *k*-means clustering analysis is utilised for the final clustering. For a given snapshot, contact vector $c_i$ ($i$=1,⋯,306) is calculated as described in the above. Then for each contact vector, the distance $D_s$ to the cluster of *clsid=s* is defined as

$$D_s = \sqrt{\sum_{i=1}^{306} [c_i - G_i(s)]^2}$$

where $G_i(s)$ is cluster centre of the cluster of *clsid=s*. The cluster centre is the **mean contact ratio** which is defined as the averaged contact data over all member of the cluster of *clsid=s*,

$$G_i(s) = \frac{1}{M_s} \sum_{j \in \Omega_s} c_i(j),$$

where $j$ is an index of contact vector data, $M_s$ is the number of data points classified as the cluster of *clsid=s* ($M_s = \sum_{j \in \Omega_s} 1$), and $\Omega_s$ is the group of $j$ (index of data points)

classified as the cluster *clsid=s*. The cluster id *clsid* to which a given data point belongs can be determined by finding the cluster giving minimum distance among distances to the 9 clusters. Table C2 shows the list of cluster centres, where residues which have mean contact ratio less than 0.1 (less than 0.05 for *clsid*=9) were omitted from the list, but this list of centres is sufficient to reproduce original classification for 99.8% of present data points. The standard errors were estimated by Jackknife method[6] based on virtual 196 samples removing each one of the 196 trajectories, recalculating cluster centres by applying *k*-means clustering for each virtual samples.

Supplementary Table C1. Comparison between hierarchical clustering and *k*-means clustering (based on hierarchical clustering with *k*=9) for total 196000 data points for all 7 ligands. Each row/column shows number of data points classified to the site by hierarchical/*k*-means clustering. 90% of classification is common for both clustering methods (shown in diagonals).

| /*k*-means<br>hierarchical | *clsid*=1 | *clsid*=2 | *clsid*=3 | *clsid*=4 | *clsid*=5 | *clsid*=6 | *clsid*=7 | *clsid*=8 | *clsid*=9 |
|---|---|---|---|---|---|---|---|---|---|
| *clsid*=1 | 26589 | 1 | 0 | 0 | 75 | 38 | 240 | 0 | 680 |
| *clsid*=2 | 1 | 19786 | 0 | 0 | 0 | 14 | 0 | 0 | 268 |
| *clsid*=3 | 0 | 0 | 15963 | 0 | 0 | 0 | 0 | 4 | 1225 |
| *clsid*=4 | 0 | 0 | 123 | 16118 | 0 | 0 | 0 | 0 | 610 |
| *clsid*=5 | 724 | 0 | 0 | 0 | 10513 | 0 | 0 | 2286 | 348 |
| *clsid*=6 | 26 | 0 | 0 | 0 | 0 | 12603 | 0 | 0 | 903 |
| *clsid*=7 | 10 | 0 | 0 | 0 | 0 | 0 | 6400 | 0 | 98 |
| *clsid*=8 | 0 | 0 | 5 | 9 | 0 | 0 | 0 | 12267 | 2083 |
| *clsid*=9 | 69 | 2947 | 1 | 440 | 972 | 3015 | 2100 | 99 | 56347 |

Supplementary Table C2. List of cluster centres (mean contact ratios). As to the correspondence between *clsid* and site name, see Supplementary information D. Residue names are listed in descending order of their mean contact ratios and residues which have mean contact ratio larger than 0.2 are shown with bold.

*clsid*=1 (site4)

| resid | mean | error | resid | mean | error | resid | mean | error |
|---|---|---|---|---|---|---|---|---|
| **Tyr154** | 0.918 | 0.023 | **Tyr118** | 0.860 | 0.043 | **Arg298** | 0.858 | 0.054 |
| **Ser123** | 0.810 | 0.053 | **Ser301** | 0.777 | 0.055 | **Ser121** | 0.756 | 0.045 |
| **Leu141** | 0.721 | 0.061 | **Asn119** | 0.590 | 0.060 | **Val297** | 0.575 | 0.065 |
| **Phe8** | 0.504 | 0.058 | **Pro122** | 0.494 | 0.045 | **Asp153** | 0.465 | 0.052 |
| **Ile152** | 0.465 | 0.057 | **Phe294** | 0.376 | 0.053 | **Gly302** | 0.306 | 0.057 |
| **Gly71** | 0.303 | 0.074 | **Asn142** | 0.292 | 0.044 | **Asp155** | 0.274 | 0.054 |
| **Gln19** | 0.265 | 0.063 | **Pro9** | 0.251 | 0.052 | **Gln69** | 0.233 | 0.063 |
| **Gly120** | 0.212 | 0.065 | Val303 | 0.173 | 0.049 | Ala70 | 0.156 | 0.049 |
| Val18 | 0.155 | 0.050 | Gln299 | 0.150 | 0.042 | Asn72 | 0.130 | 0.042 |
| Gly143 | 0.126 | 0.028 | Met17 | 0.103 | 0.039 | Cys300 | 0.102 | 0.037 |

*clsid*=2 (site1, the active site)

| resid | mean | error | resid | mean | error | resid | mean | error |
|---|---|---|---|---|---|---|---|---|
| **Gln189** | 0.917 | 0.019 | **Met49** | 0.881 | 0.031 | **Hie41** | 0.877 | 0.032 |
| **Met165** | 0.802 | 0.047 | **Glu166** | 0.663 | 0.059 | **Asn142** | 0.643 | 0.050 |
| **Thr25** | 0.615 | 0.064 | **Cys145** | 0.609 | 0.054 | **Ser46** | 0.579 | 0.054 |
| **Hie164** | 0.570 | 0.044 | **Leu27** | 0.532 | 0.066 | **Asp187** | 0.512 | 0.064 |
| **Gly143** | 0.505 | 0.064 | **Pro168** | 0.440 | 0.072 | **Cys44** | 0.401 | 0.061 |
| **Thr190** | 0.395 | 0.063 | **Arg188** | 0.377 | 0.061 | **Leu50** | 0.374 | 0.059 |
| **Leu167** | 0.357 | 0.064 | **Thr26** | 0.333 | 0.060 | **Ala191** | 0.332 | 0.069 |
| **Thr45** | 0.294 | 0.046 | **Gln192** | 0.275 | 0.057 | **Tyr54** | 0.253 | 0.057 |
| **Thr24** | 0.208 | 0.045 | Ser1 | 0.158 | 0.047 | Ser144 | 0.140 | 0.046 |
| Hie163 | 0.133 | 0.036 | Thr304 | 0.120 | 0.031 | Asn119 | 0.116 | 0.040 |
| Val303 | 0.112 | 0.026 | Phe305 | 0.105 | 0.027 | Val186 | 0.102 | 0.030 |

*clsid*=3 (site2)

| resid | mean | error | resid | mean | error | resid | mean | error |
|---|---|---|---|---|---|---|---|---|
| **Leu272** | 0.948 | 0.014 | **Gly275** | 0.939 | 0.019 | **Leu286** | 0.938 | 0.015 |
| **Met276** | 0.903 | 0.021 | **Tyr237** | 0.883 | 0.035 | **Leu287** | 0.852 | 0.033 |
| **Tyr239** | 0.752 | 0.045 | **Gly278** | 0.730 | 0.075 | **Leu271** | 0.715 | 0.042 |
| **Ala285** | 0.694 | 0.071 | **Asn277** | 0.646 | 0.078 | **Thr280** | 0.445 | 0.069 |
| **Asn274** | 0.393 | 0.072 | **Gln273** | 0.335 | 0.070 | **Arg279** | 0.330 | 0.065 |
| **Asn238** | 0.325 | 0.062 | **Thr199** | 0.261 | 0.069 | **Lys236** | 0.219 | 0.056 |
| **Asp197** | 0.207 | 0.071 | Thr198 | 0.175 | 0.064 | Arg131 | 0.119 | 0.054 |
| Gly283 | 0.111 | 0.039 | | | | | | |

*clsid*=4 (site5)

| resid | mean | error | resid | mean | error | resid | mean | error |
|---|---|---|---|---|---|---|---|---|
| **Leu220** | 0.920 | 0.042 | **Arg222** | 0.814 | 0.046 | **Asn221** | 0.753 | 0.046 |
| **Arg217** | 0.724 | 0.073 | **Ile259** | 0.611 | 0.119 | **Trp218** | 0.522 | 0.115 |
| **Gly258** | 0.355 | 0.099 | **Phe219** | 0.347 | 0.122 | **Glu270** | 0.332 | 0.123 |
| **Thr257** | 0.332 | 0.098 | **Asp263** | 0.301 | 0.070 | **Asn274** | 0.295 | 0.118 |
| **Phe305** | 0.294 | 0.090 | **Val212** | 0.230 | 0.087 | **Leu271** | 0.228 | 0.104 |
| **Arg279** | 0.219 | 0.088 | Phe223 | 0.173 | 0.047 | Gln306 | 0.170 | 0.057 |
| Ala260 | 0.144 | 0.072 | Ile213 | 0.129 | 0.070 | Gln256 | 0.124 | 0.061 |
| Ser267 | 0.117 | 0.036 | Gly275 | 0.112 | 0.060 | Ala255 | 0.111 | 0.068 |
| Asn277 | 0.107 | 0.065 | | | | | | |

*clsid*=5

| resid | mean | error | resid | mean | error | resid | mean | error |
|---|---|---|---|---|---|---|---|---|
| **Phe294** | 0.920 | 0.078 | **Ile249** | 0.831 | 0.098 | **Gln110** | 0.778 | 0.112 |
| **Asp153** | 0.551 | 0.133 | **Pro293** | 0.534 | 0.088 | **Val297** | 0.534 | 0.164 |
| **Asn151** | 0.512 | 0.123 | **Thr292** | 0.505 | 0.134 | **Pro252** | 0.465 | 0.142 |
| **Gln107** | 0.452 | 0.157 | **Val104** | 0.426 | 0.111 | **Arg298** | 0.405 | 0.148 |
| **Ser158** | 0.382 | 0.107 | **Hie246** | 0.378 | 0.161 | **Ile106** | 0.366 | 0.095 |
| **Gly109** | 0.353 | 0.141 | **Val202** | 0.337 | 0.144 | **Ile152** | 0.323 | 0.109 |
| **Asp245** | 0.302 | 0.111 | **Pro108** | 0.295 | 0.135 | **Tyr154** | 0.280 | 0.131 |
| **Asn203** | 0.223 | 0.089 | **Ser301** | 0.218 | 0.129 | **Thr111** | 0.215 | 0.051 |
| **Ile200** | 0.209 | 0.115 | Phe8 | 0.159 | 0.077 | Cys160 | 0.155 | 0.064 |
| Arg105 | 0.154 | 0.048 | Thr243 | 0.141 | 0.083 | Asp248 | 0.141 | 0.034 |
| Lys102 | 0.134 | 0.037 | Leu253 | 0.120 | 0.063 | | | |

*clsid*=6

| resid | mean | error | resid | mean | error | resid | mean | error |
|---|---|---|---|---|---|---|---|---|
| **Leu67** | 0.929 | 0.055 | **Gln74** | 0.782 | 0.053 | **Thr21** | 0.687 | 0.097 |
| **Gly23** | 0.573 | 0.092 | **Arg76** | 0.572 | 0.100 | **Hid64** | 0.567 | 0.090 |
| **Asn65** | 0.500 | 0.095 | **Leu75** | 0.465 | 0.096 | **Phe66** | 0.404 | 0.076 |
| **Gln69** | 0.356 | 0.079 | **Thr26** | 0.350 | 0.090 | **Gln19** | 0.314 | 0.083 |
| **Thr24** | 0.301 | 0.075 | **Cys22** | 0.246 | 0.054 | **Asn119** | 0.204 | 0.065 |
| **Val73** | 0.203 | 0.088 | Val68 | 0.191 | 0.058 | Asn72 | 0.160 | 0.043 |
| Val20 | 0.139 | 0.054 | Thr93 | 0.136 | 0.078 | Val77 | 0.135 | 0.057 |
| Asn63 | 0.117 | 0.058 | Asp92 | 0.113 | 0.071 | Gly71 | 0.110 | 0.042 |

*clsid*=7 (site3)

| resid | mean | error | resid | mean | error | resid | mean | error |
|---|---|---|---|---|---|---|---|---|
| **Pro96** | 0.912 | 0.034 | **Trp31** | 0.842 | 0.061 | **Lys97** | 0.827 | 0.065 |
| **Val73** | 0.803 | 0.076 | **Ala70** | 0.757 | 0.068 | **Lys12** | 0.702 | 0.082 |
| **Pro99** | 0.619 | 0.081 | **Gly15** | 0.613 | 0.099 | **Thr93** | 0.580 | 0.108 |
| **Leu75** | 0.568 | 0.101 | **Thr98** | 0.514 | 0.077 | **Gly11** | 0.479 | 0.089 |
| **Gly71** | 0.465 | 0.090 | **Asn95** | 0.402 | 0.104 | **Glu14** | 0.379 | 0.082 |
| **Asn72** | 0.360 | 0.064 | **Met17** | 0.273 | 0.101 | **Cys16** | 0.251 | 0.112 |
| Lys100 | 0.199 | 0.053 | Val18 | 0.188 | 0.089 | Pro122 | 0.164 | 0.064 |
| Gln74 | 0.136 | 0.055 | Ser121 | 0.124 | 0.042 | Ala94 | 0.123 | 0.036 |
| Asp155 | 0.105 | 0.045 | | | | | | |

*clsid*=8

| resid | mean | error | resid | mean | error | resid | mean | error |
|---|---|---|---|---|---|---|---|---|
| **Met235** | 0.857 | 0.062 | **Pro241** | 0.807 | 0.065 | **Leu232** | 0.679 | 0.103 |
| **Asn231** | 0.653 | 0.143 | **Asn228** | 0.556 | 0.134 | **Thr243** | 0.501 | 0.102 |
| **Leu227** | 0.383 | 0.104 | **Leu242** | 0.380 | 0.093 | **Glu240** | 0.343 | 0.108 |
| **Thr198** | 0.332 | 0.100 | **Gln244** | 0.310 | 0.091 | **Lys236** | 0.291 | 0.128 |
| **Asn238** | 0.257 | 0.088 | **Tyr239** | 0.218 | 0.058 | Thr196 | 0.195 | 0.085 |
| Pro132 | 0.160 | 0.125 | Pro108 | 0.154 | 0.126 | Hie246 | 0.129 | 0.096 |
| Ile200 | 0.121 | 0.108 | Ala234 | 0.114 | 0.039 | | | |

*clsid*=9

| resid | mean | error | resid | mean | error | resid | mean | error |
|---|---|---|---|---|---|---|---|---|
| Ala255 | 0.163 | 0.035 | Pro184 | 0.131 | 0.028 | Pro252 | 0.114 | 0.026 |
| Gln256 | 0.114 | 0.027 | Arg105 | 0.110 | 0.025 | Val186 | 0.106 | 0.024 |
| Asn180 | 0.103 | 0.024 | Asn84 | 0.101 | 0.025 | Thr190 | 0.100 | 0.019 |
| Gly183 | 0.099 | 0.025 | Gly258 | 0.098 | 0.026 | Phe181 | 0.096 | 0.025 |
| Pro168 | 0.089 | 0.021 | Gly251 | 0.086 | 0.022 | Thr304 | 0.086 | 0.019 |
| Gly302 | 0.085 | 0.023 | Ala191 | 0.084 | 0.017 | Phe134 | 0.079 | 0.023 |
| Val303 | 0.077 | 0.019 | Pro52 | 0.077 | 0.020 | Ser254 | 0.076 | 0.023 |
| Arg188 | 0.076 | 0.016 | Gln189 | 0.070 | 0.018 | Ala260 | 0.069 | 0.022 |
| Phe305 | 0.069 | 0.015 | Gln192 | 0.068 | 0.014 | Ser81 | 0.066 | 0.022 |
| Leu50 | 0.064 | 0.015 | Asn53 | 0.063 | 0.018 | Gln306 | 0.063 | 0.013 |
| Ser301 | 0.062 | 0.019 | Hid80 | 0.062 | 0.022 | Asn214 | 0.062 | 0.017 |
| Glu55 | 0.061 | 0.019 | Phe185 | 0.061 | 0.013 | Thr169 | 0.061 | 0.016 |
| Ile59 | 0.059 | 0.013 | Asp248 | 0.058 | 0.015 | Met82 | 0.057 | 0.018 |
| Thr257 | 0.053 | 0.017 | Val247 | 0.053 | 0.018 | Cys85 | 0.052 | 0.018 |
| Arg40 | 0.051 | 0.015 | Ser1 | 0.051 | 0.015 | Lys88 | 0.050 | 0.020 |
| Asn51 | 0.050 | 0.012 | Ala193 | 0.050 | 0.011 | | | |

## Supplementary information D. Robustness of the clustering analysis.

[TOP]

In order to see the results of the hierarchical clustering are sufficiently robust and consistent, the clusters detected at level 9 with four different conditions are shown in the Figs. D1-2, where, for each cluster, residues having mean contact ratio above 0.8(red), 0.6(orange), 0.4(yellow), 0.2(white) are drawn with the surface plot view, except the clusters shown at the bottom row in Fig. D2 which show residues with ratio above 0.08 (red) (because maximum values of the mean contact ratio were below 0.2, i.e., these clusters correspond to “not yet classified” contacts). Here, the mean contact ratio for each cluster was defined as the averaged contact vector data over the data points classified to the cluster (see also Supplementary information C). We examined two variations for the threshold lengths $d$=0.35nm and $d$=0.3nm to judge contact between a ligand and a residue. We also examined two cases of the trajectory lengths: in one case, whole frames (1-1000) in each trajectory were used, and in another case, only later-half frames (501-1000) in each trajectory used for the clustering analysis (total 98,000 frames were analysed in the latter case). The figures clearly show that these four clustering analyses gave similar results, although the cluster identifier and the classified order were somewhat different. In this paper, we adopted the classification with $d$=0.35nm using whole frames (schematic figures shown on left-most columns in Figs. D1-2.). The clusters 1, 2, 3, 4, and 7 correspond to site 4, the active site, sites 2, 5, and 3, respectively. We focused only these sites and treated the other clusters 5, 6, 8, and 9 as merely the “other” site.

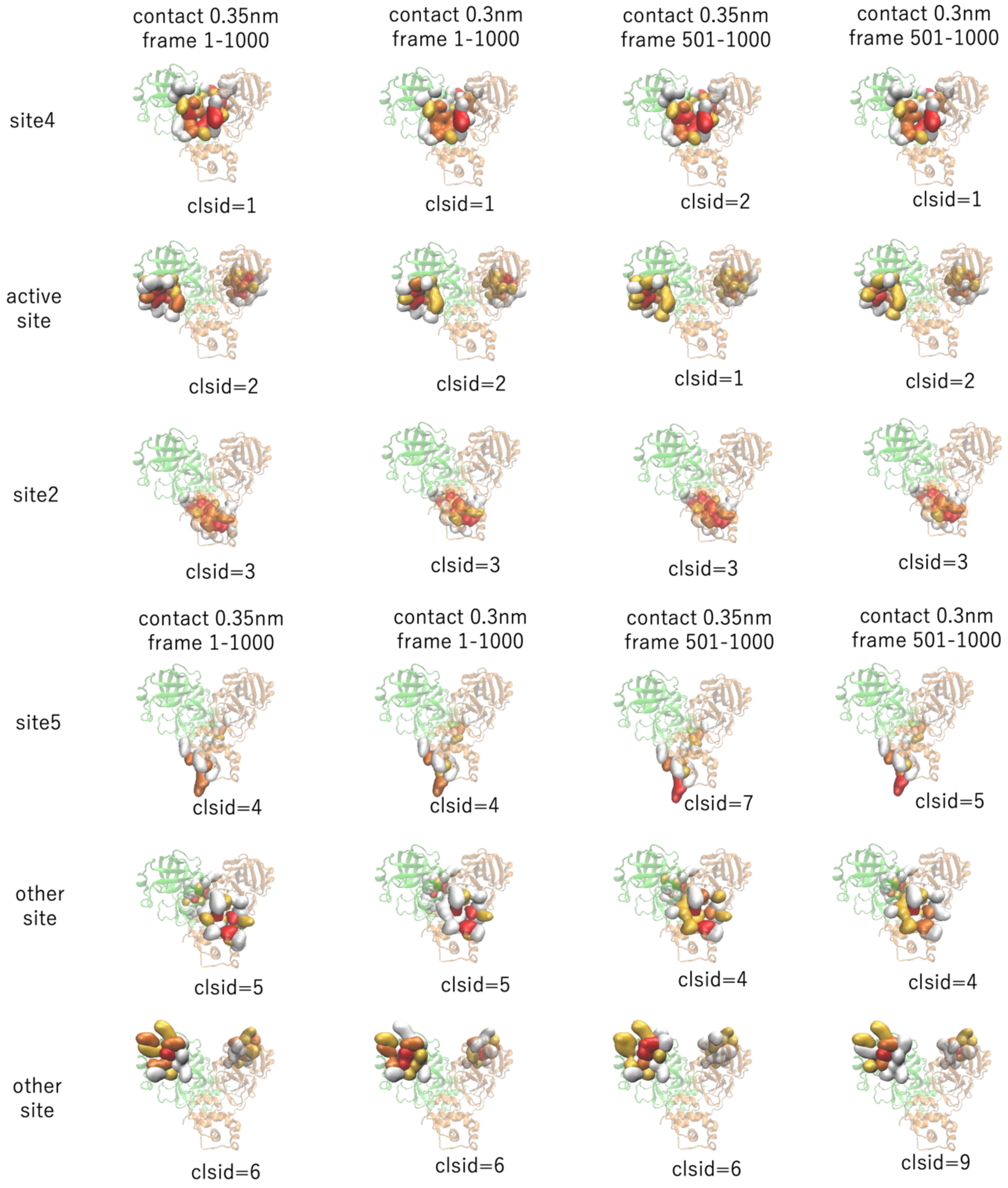


Supplementary Figure D1. Comparison of top nine clusters detected under four different conditions. Each row shows similar clusters obtained for four different conditions, and also the corresponding site label (used in the manuscript) shown leftmost column.

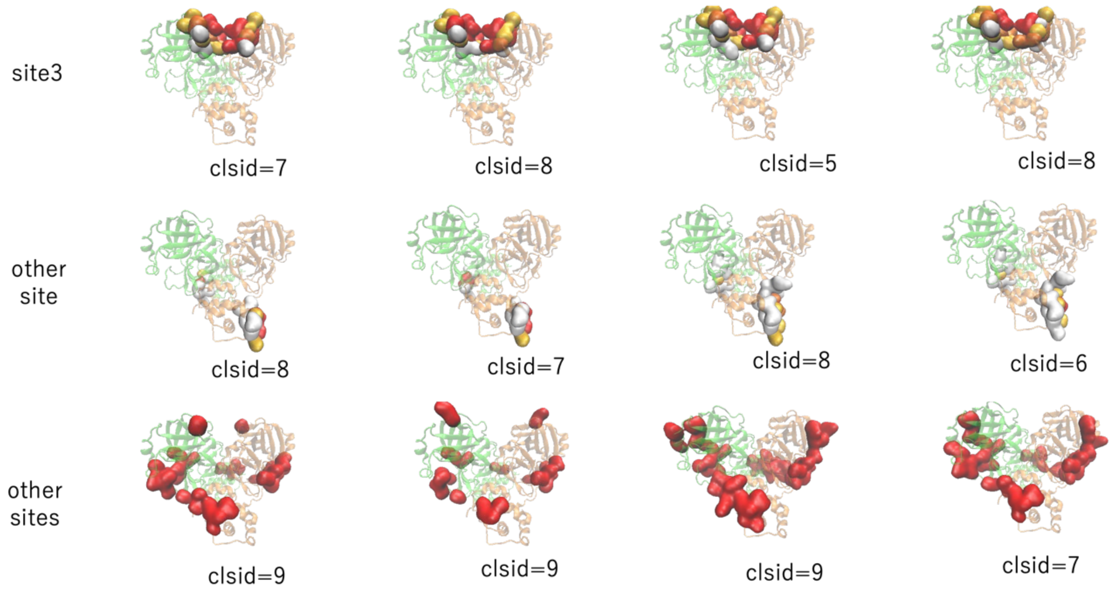


Supplementary Figure D2. (continued from D1) Comparison of top nine clusters detected under four different conditions.

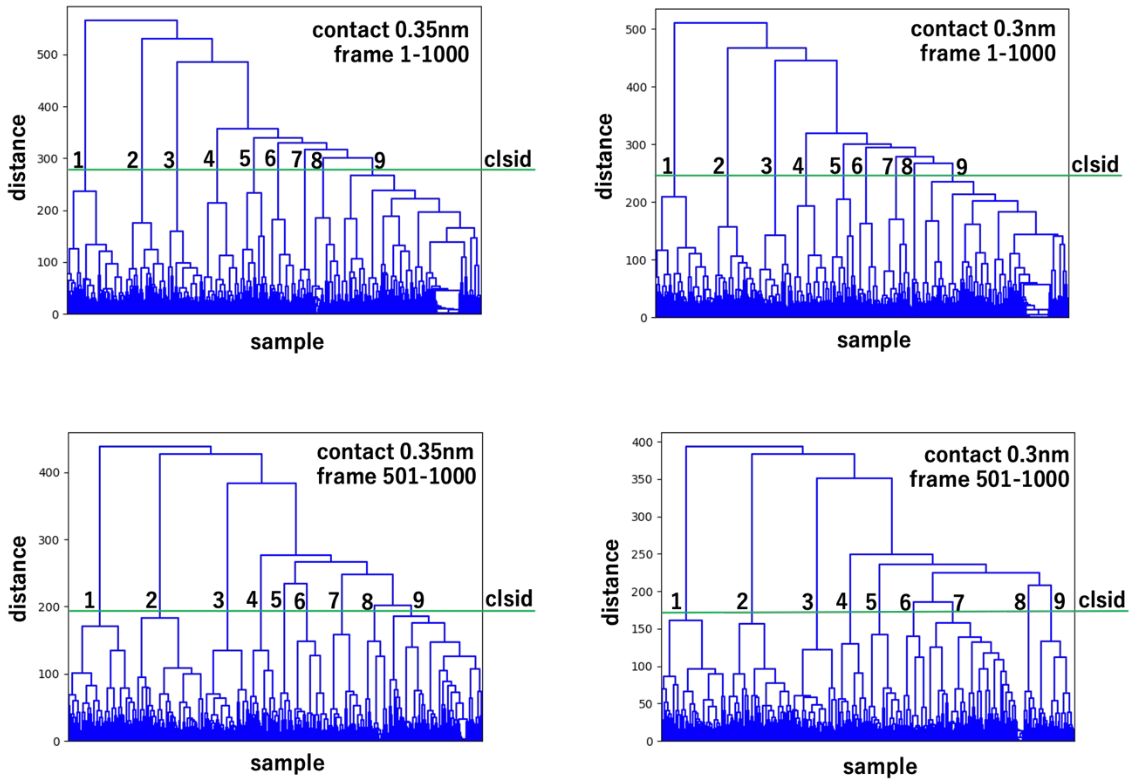


Supplementary Figure D3. Dendrograms for clustering analyses under four different conditions. Top nine clusters detected for each condition are shown.

## Supplementary information E. Time series of contact positions of ligands and MM-GB/SA binding free energies. [TOP]

Time series of contact positions of ligands and binding free energies estimated by the molecular mechanics generalized−Born surface area (MM-GB/SA) method are shown in Figs. E1-7 for 28 trajectories of each seven ligand. Rows 1 (top) - 4 (bottom) correspond to the initial ligand locations A-D (Supplementary Fig. B1), and the seven columns correspond to the runs with the differently randomised initial velocities. The classification of the positions has been assigned by the *k*-means clustering analysis described in the main text and supplementary information C. Each trajectory is 200-ns long sampled every 0.2 ns and the green line shows the contact positions (the left axes). The blue lines were smoothed ones by taking the majority for 71 continuous data points (± 35 points). The red and orange lines (the right axes) are binding free energies that were calculated by the MM-GB/SA method and averaged over 15 continuous data points (± 7 points). The red and orange parts of the lines indicate that the solvent-accessible surface area (SASA) of each ligand were below and above 50 % of the SASA of the solvated ligand, respectively. Thus, a ligand is expected to have the stronger binding to the sites at the red parts. The SASAs of the solvated ligands were estimated from the last 2.5 ns of the MD simulations of 5 ns (Supplementary Table E1). Figure E8 shows the time series of binding free energies averaged over 28 trajectories. The standard deviations were only depicted for ritonavir, but they are at similar levels for all ligands. The deviations were large because they were averaged over different states. Table E2 shows the occupation ratio of each site for last 100 ns averaged over 28 trajectories of each ligand.

The MM-GB/SA calculations were carried out using MMPBSA.py program[7] of AmberTools18. For the MM-GB/SA calculations, the binding free energy, $\Delta G$, was calculated using the coordinates of the receptor-ligand, receptor only and ligand only systems extracted from the same MD trajectory of receptor-ligand system. The binding free energy can be obtained according to the following equations.

$$\Delta G = \Delta E_{\mathrm{bind}}^{\mathrm{gas}} + \Delta G_{\mathrm{bind}}^{\mathrm{solv}} - T\Delta S_{\mathrm{bind}}^{\mathrm{gas}}$$

$$\Delta E_{\mathrm{bind}}^{\mathrm{gas}} = E_{\mathrm{receptor-ligand}}^{\mathrm{gas}} - E_{\mathrm{receptor}}^{\mathrm{gas}} - E_{\mathrm{ligand}}^{\mathrm{gas}}$$

$$\Delta G_{\mathrm{bind}}^{\mathrm{solv}} = G_{\mathrm{receptor-ligand}}^{\mathrm{solv}} - G_{\mathrm{receptor}}^{\mathrm{solv}} - G_{\mathrm{ligand}}^{\mathrm{solv}}$$

$$\Delta G_{\mathrm{bind}}^{\mathrm{solv}} = \Delta G_{\mathrm{bind}}^{\mathrm{solv,polar}} + \Delta G_{\mathrm{bind}}^{\mathrm{solv,nonpolar}}$$

Here, $\Delta E_{\text{bind}}^{\text{gas}}$ indicates the gas-phase interaction energy between the receptor and the ligand, which is the difference between the gas-phase potential energy of the receptor-ligand system and the sum of the gas-phase potential energies for the receptor only system and for the ligand only system. The term $\Delta G_{\text{bind}}^{\text{solv}}$ is the solvation energy difference upon protein-ligand association and is computed as the sum of polar and nonpolar terms ($\Delta G_{\text{bind}}^{\text{solv,polar}}$ + $\Delta G_{\text{bind}}^{\text{solv,nonpolar}}$). The term $\Delta G_{\text{bind}}^{\text{solv,polar}}$ can be calculated numerically by solving the generalized Born model developed by Onufriev et al[8]. The term $\Delta G_{\text{bind}}^{\text{solv,nonpolar}}$ can be calculated using the following equation.

$$\Delta G_{\text{bind}}^{\text{solv,nonpolar}} = \gamma SASA + \beta$$

Here, *SASA* represents the solvent-accessible surface area, calculated using the linear combinations of pairwise overlaps method[9], and the values for the γ and $\beta$ were set to 0.005 kcal/mol·Å$^2$ and 0 kcal/mol. In this calculation, the entropic term $T\Delta S_{\text{bind}}^{\text{gas}}$ was not taken into consideration.

Supplementary Table E1. The Solvent-Accessible Surface Area (SASA) of each ligand. The standard deviations are shown in the parenthesis. The values were estimated by separate 5 ns MD simulations of the systems of the solvated ligands. The last half (2.5 ns) of each trajectory has been used.

| **Ligand** | **SASA (nm$^2$)** |
|---|---|
| Darunavir | 7.21 (0.45) |
| Indinavir | 8.80 (0.91) |
| Lopinavir | 8.65 (1.48) |
| Nelfinavir | 8.05 (1.07) |
| Ritonavir | 8.68 (0.22) |
| Saquinavir | 8.15 (0.84) |
| Tipranavir | 8.13 (1.19) |

Supplementary Table E2. The occupation ratio (in percent) of each site for last 100 ns trajectories of 200 ns simulations. All 28 trajectories for each ligand are used.

| Ligand | Active site | Site 2 | Site 3 | Site 4 | Site 5 | Others |
|---|---|---|---|---|---|---|
| Darunavir | 7.0 | 11.3 | 0.3 | 10.8 | 4.6 | 65.9 |
| Indinavir | 28.1 | 5.5 | 1.6 | 20.4 | 1.5 | 42.9 |
| Lopinavir | 5.7 | 12.7 | 17.9 | 3.6 | 14.2 | 45.9 |
| Nelfinavir | 16.0 | 9.1 | 0.0 | 26.5 | 10.9 | 37.5 |
| Ritonavir | 20.1 | 17.8 | 3.7 | 14.8 | 6.4 | 37.2 |
| Saquinavir | 12.6 | 16.8 | 1.7 | 21.1 | 7.3 | 40.5 |
| Tipranavir | 16.5 | 0.0 | 4.0 | 16.0 | 15.5 | 48.0 |

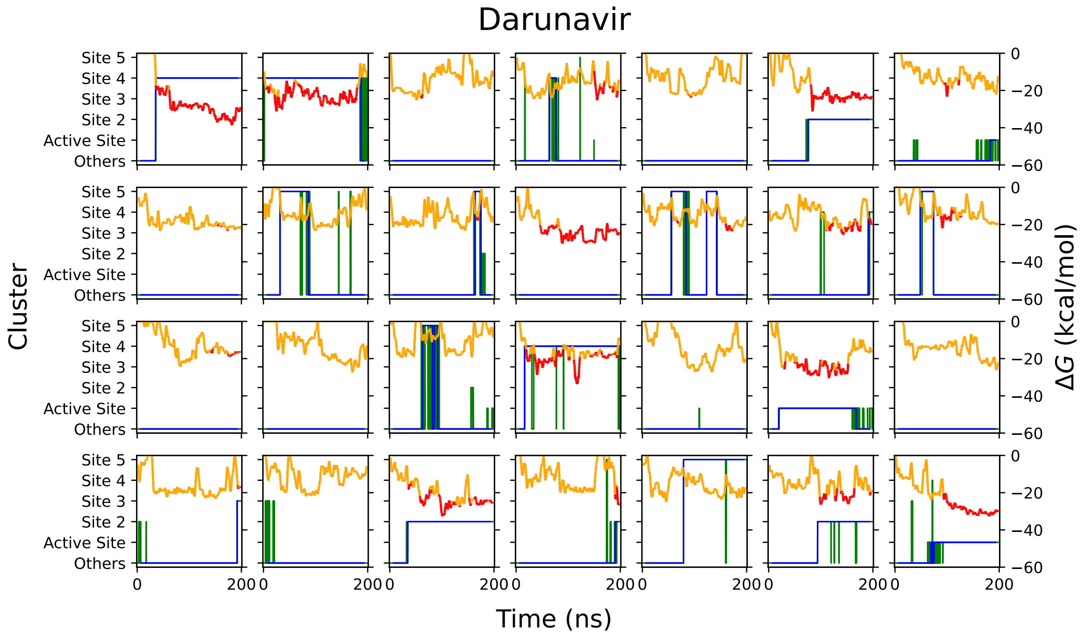


Supplementary Figure E1. Time series of contact position and free energies of darunavir.

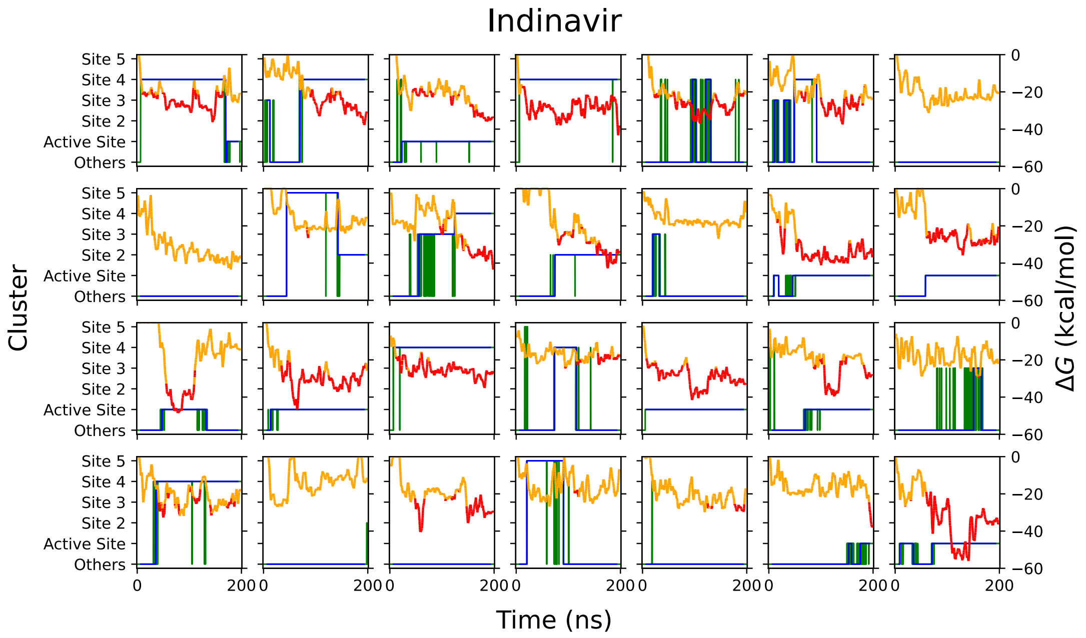


Supplementary Figure E2. Time series of contact position and free energies of indinavir.

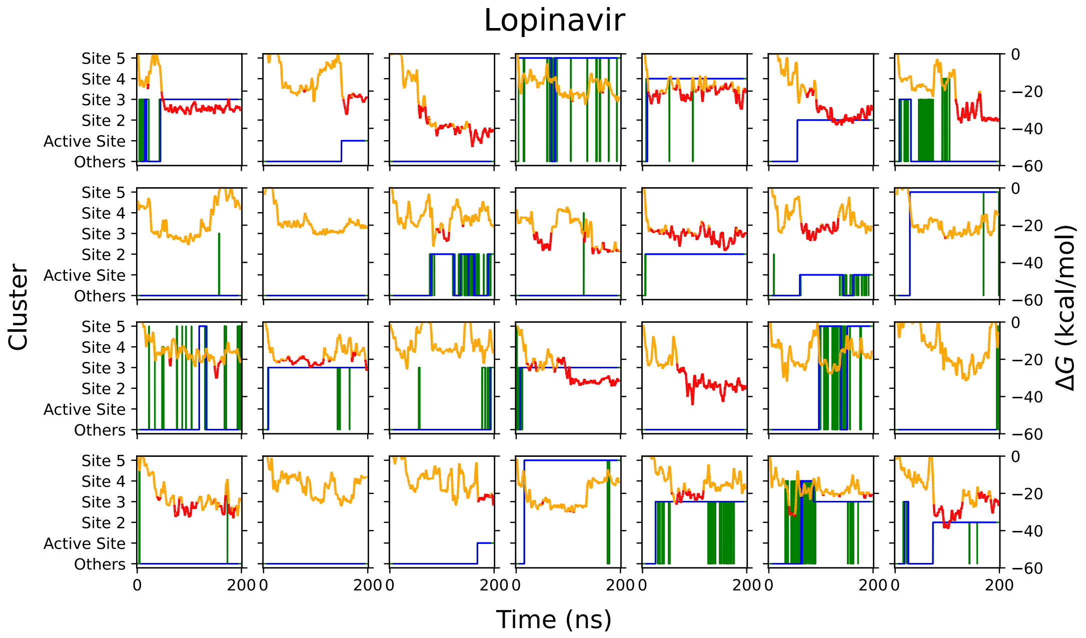


Supplementary Figure E3. Time series of contact position and free energies of lopinavir.

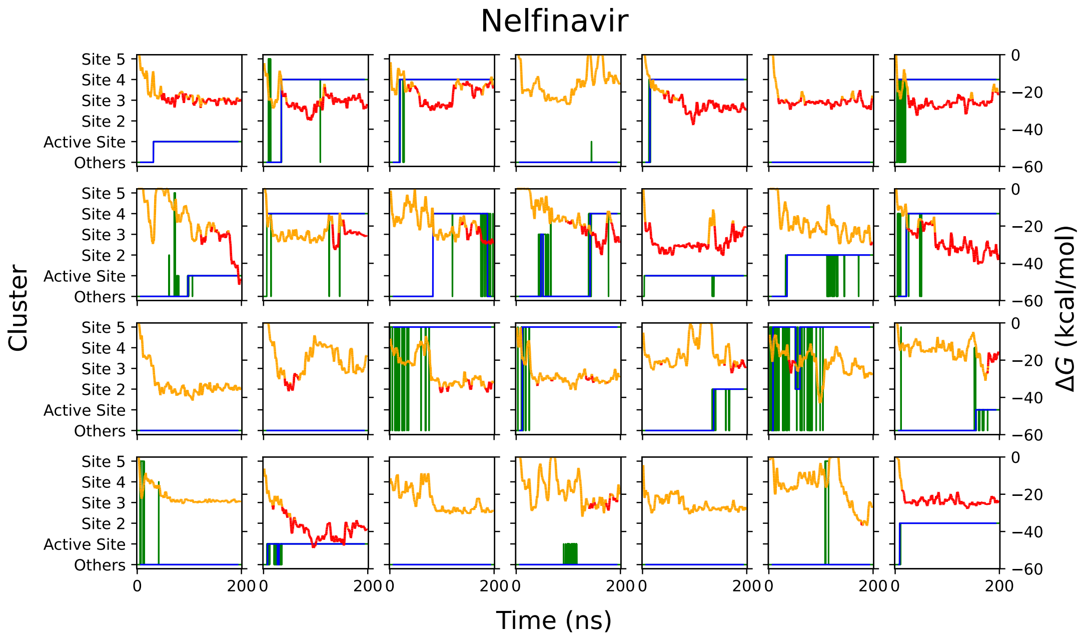


Supplementary Figure E4. Time series of contact position and free energies of nelfinavir.

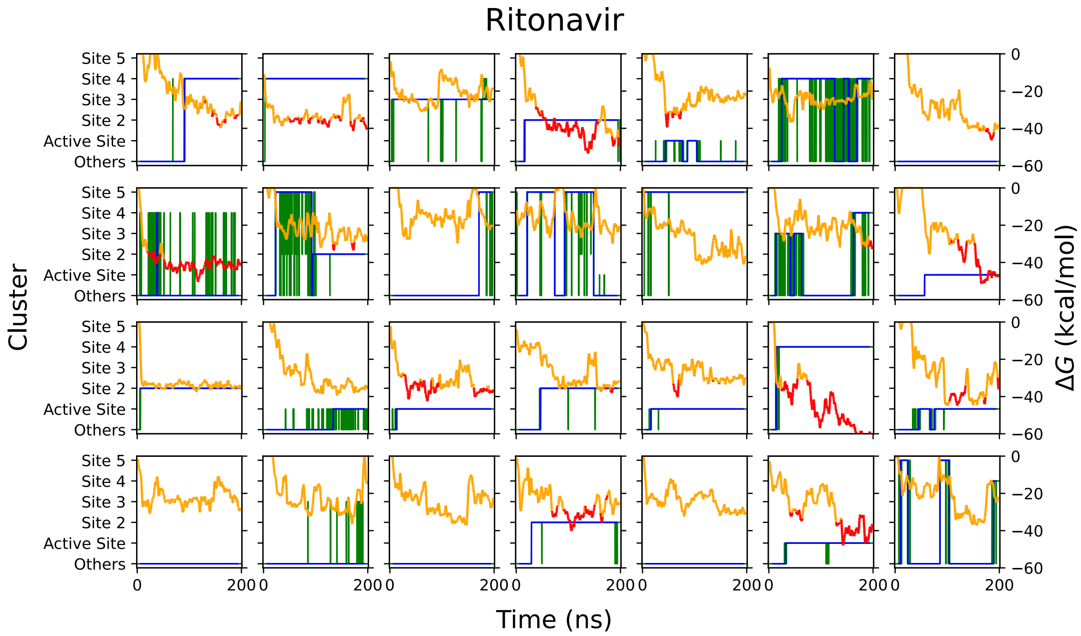


Supplementary Figure E5. Time series of contact position and free energies of ritonavir.

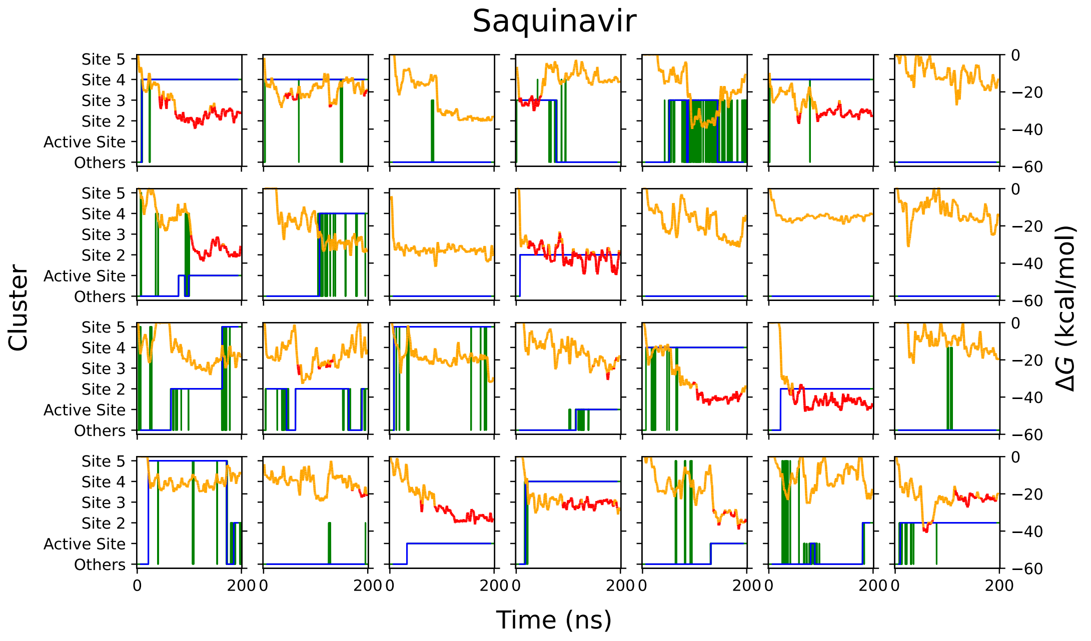


Supplementary Figure E6. Time series of contact position and free energies of saquinavir.

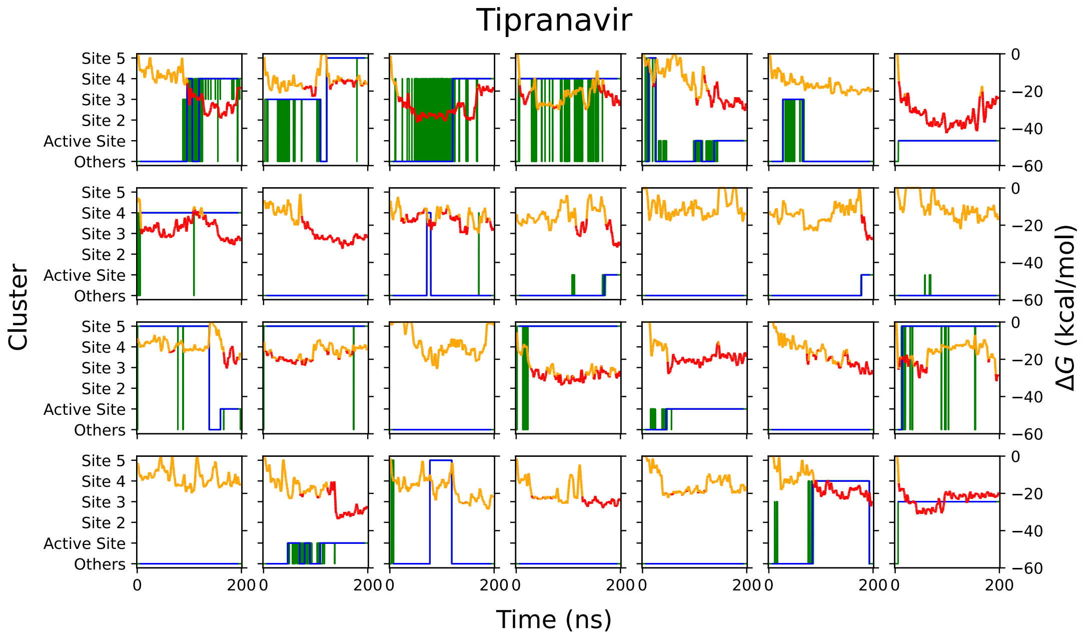


Supplementary Figure E7. Time series of contact position and free energies of tipranavir.

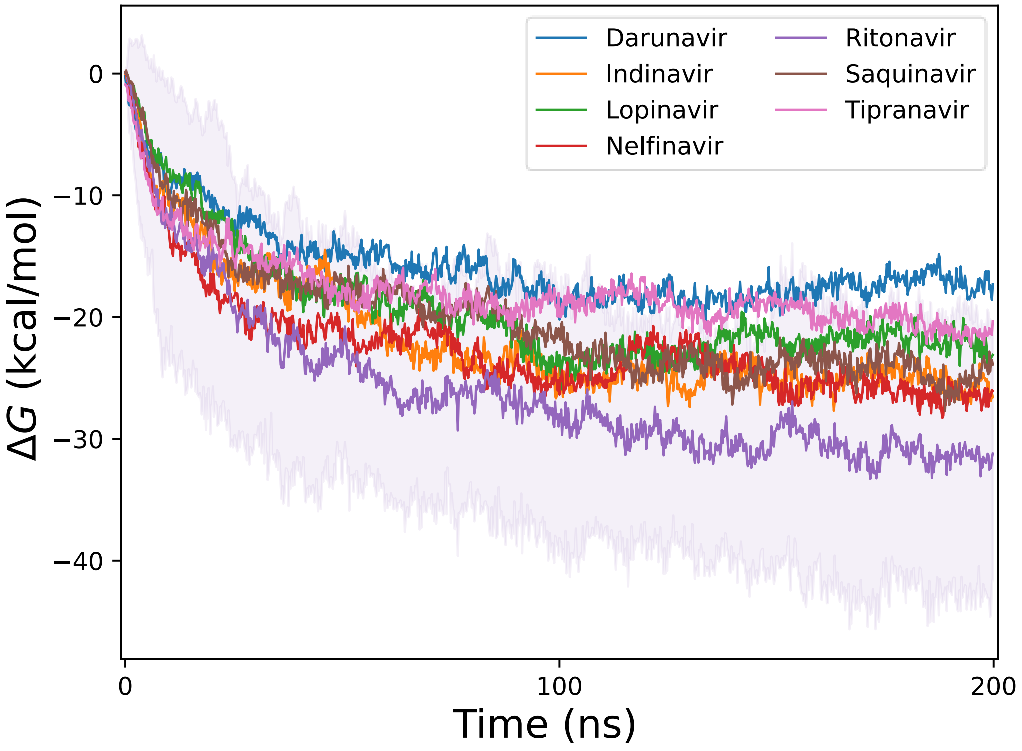


Supplementary Figure E8. Time series of average free energies. The standard deviation was shown only for ritonavir.

## Supplementary information F. Time course of occupation ratio in the classified binding sites. [TOP]

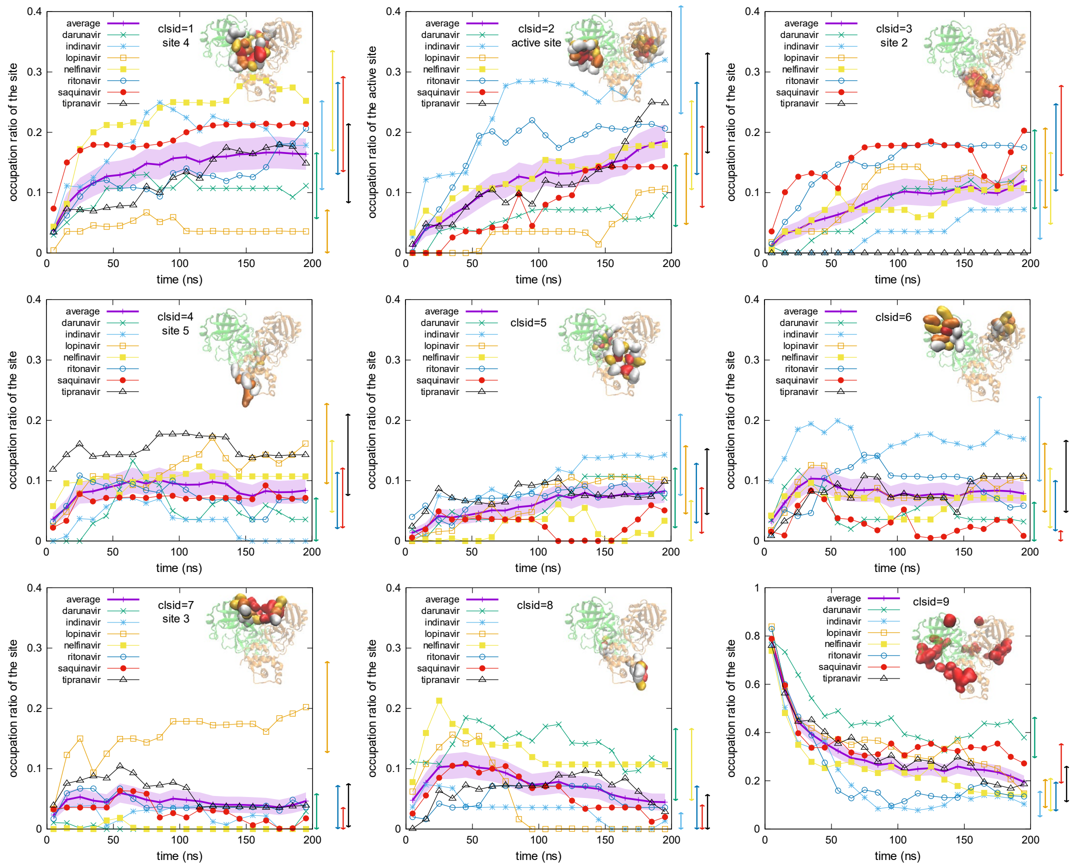


Supplementary Figure F1. The time course of occupation ratio in the nine classified binding sites in the clustering analysis at level 9. Each figure shows the mean occupation ratios averaged in every 10 ns time span and over 28 trajectories for ligands. The averaged time courses over all seven ligands are also shown with thick lines (standard error with shaded area). Standard error for final 10 ns of each ligand is shown at the right side of each graph. The fast decay observed in the 9-th figure shows ligands made a quick contact to protein surface. The time courses of the average over seven ligands show that contacts to first 3 clusters, which correspond to site 4, active site, and site 2, were major and increased in time.

***Standard error estimation:***

The standard errors were estimated by using Jackknife method[6] based on virtual samples prepared by removing one of the trajectories (as a result, we have treated 28 virtual samples for each ligand and 196 virtual samples for whole average). We have

plotted shaded area for the averaged curves, and standard error for last 10ns average for each ligand at the right side of the graphs.

More explicitly, let $i$ denote index of the trajectory ($i = 1, \cdots, N$), $X$ denote the mean of $x_i$ as

$$X = \frac{1}{N}\sum_i x_i$$

In case of occupation ratio, $x_i$ corresponds to the ratio classified to the specified *clsid* in 50 data points in 10 ns (50 x 200 ps) time span of i-th trajectory. X corresponds to the averages over 28 (or 196) trajectories for each specified *clsid*, which is shown in Fig. F1 and Fig. 3 in the main text. In order to estimate the standard error of $X$, let introduce virtual sample removing $j$-th trajectory ($j = 1, \cdots, N$) as

$$z_j = \frac{1}{N-1}\sum_{i \neq j} x_i$$

Then the standard error of $X$ is estimated[6] as

$$\sqrt{\frac{N-1}{N}\sum_j \left(z_j - X\right)^2}$$

## Supplementary information G. Negative control simulation infrequent binding to the active site. [TOP]

In the binding simulation of 7 HIV drugs shown in the main text, 7 drugs bound to the active site at least twice in 28 simulation runs. One may wonder how different frequency of binding can be observed for any other drugs. We here report additional simulation using lamivudine triphosphate (Fig. G1(a)), which showed infrequent access to the active site in contrast to frequent binding of HIV drugs to the active site. We think this observation can be a negative control simulation and also suggests importance of the binding simulation reported in the main text.

In the same manner with HIV simulations, we prepared four initial locations of the ligand (Fig. G1(b)) and 14-fold simulation runs with differently randomized initial velocity (total 56 simulation runs) were performed. Here twice runs were performed for better statistics. Figure G2 shows contact map for lamivudine triphosphate binding simulation, shown in similar way of Fig. 2 in the main text. Figure G3 shows the time course of occupation ratio in the active site, which clearly shows infrequent access to the active site. Figure G4 shows the time course of occupation ratio in the nine classified binding sites. The site for each snapshot was obtained as the nearest cluster for the contact vector of each snapshot using 9 cluster centres determined with 7 HIV contact data in Supplementary information C.

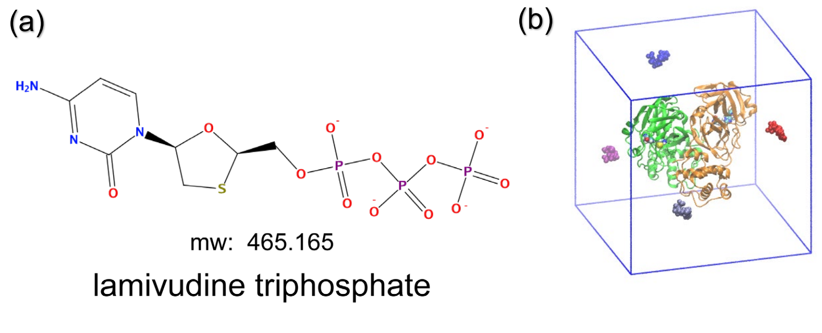


Supplementary Figure G1. (a) Structure of lamivudine triphosphate. Numerals indicate molecular weight. (b) Four initial locations of lamivudine triphosphate. A (blue), B (red), C (grey), and D (purple).

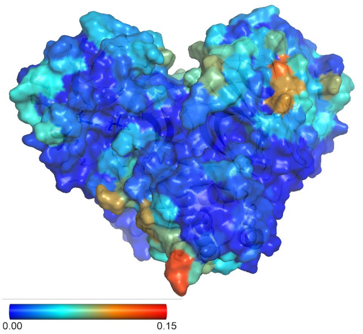


Supplementary Figure G2. Heat maps of the contact frequencies for lamivudine triphosphate shown with the same manner in Fig. 2(a). The active site location at left upper was infrequently visited, while the site 5 (*clsid*=4) at lower was frequently visited. New site at right upper (just backside of the active site) was also frequently visited, which was classified in *clsid*=9.

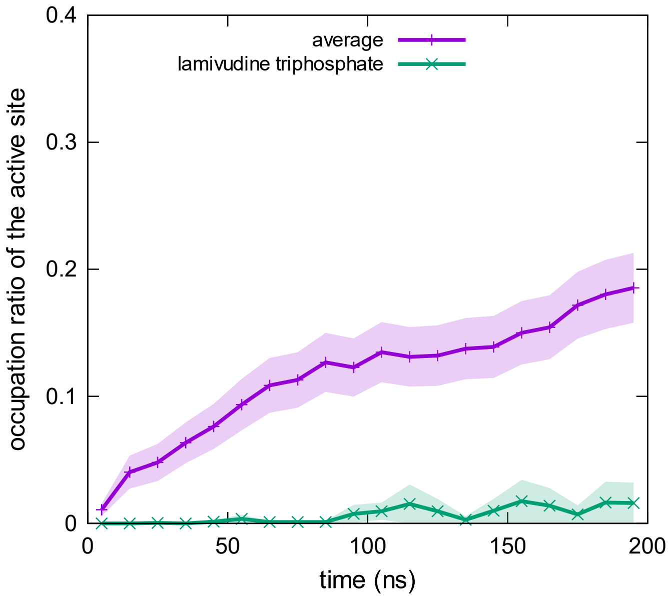


Supplementary Figure G3. Time course of occupation ratio at the active sites for lamivudine triphosphate shown with the same manner in Fig.3 (standard error shown with shaded area). For reference, the averages for 7 HIV drugs also shown.

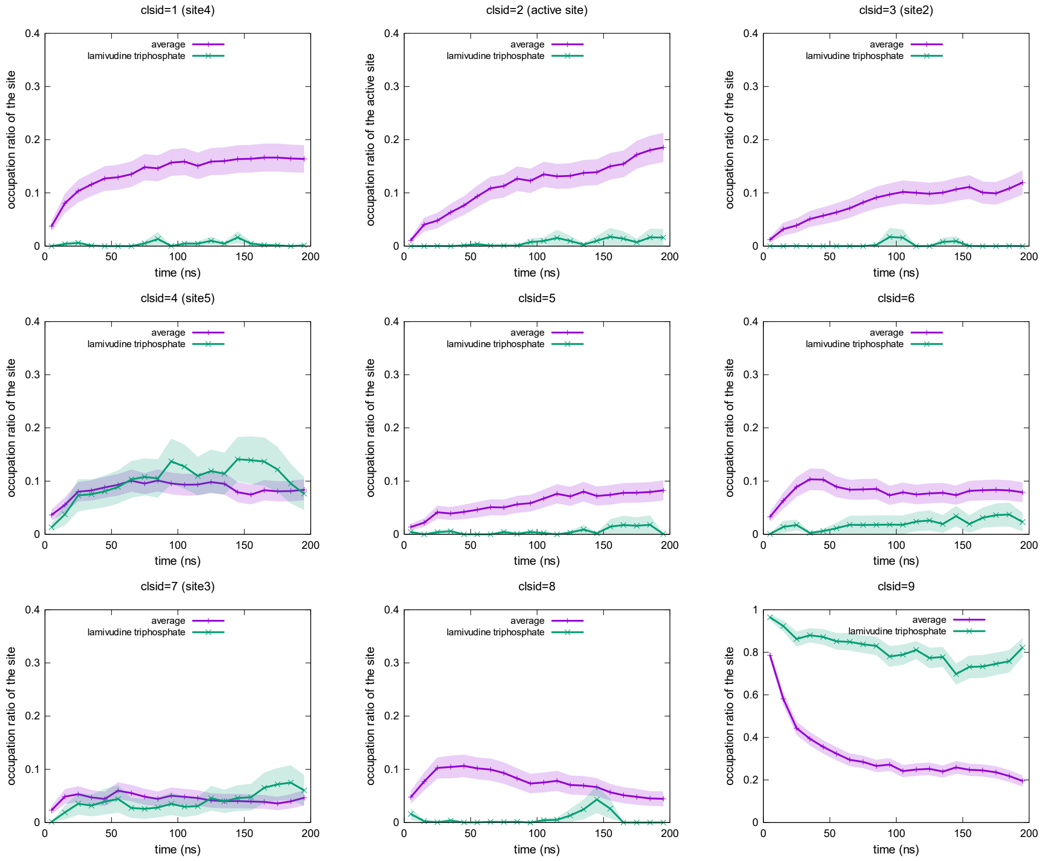


Supplementary Figure G4. The time course of occupation ratio for lamivudine triphosphate in the nine classified binding sites. For reference, the averages for 7 HIV drugs also shown.

## Supplementary information H. MM-GB/SA free energy for 1 μs trajectories. [TOP]

To observe the dynamics of ligands in the active site, 23 simulations have been extended for 1 μs. Table H1 summarises the extended simulations and the MM-GB/SA binding free energies. The extensions were chosen arbitrary from the simulations in which the ligands contacted with the active site at 200 ns. A simulation index is expressed by initial ligand locations (A-D) in Supplementary Fig. B1 and a column number in Supplementary Figs. E1-7. In the three of total 23 simulations, unbinding events from the active site were observed. These three simulations (indices shown in parenthesis in the column "simulation index" of Table H1) were excluded from the calculation of the average and the minimum binding free energies, i.e. the statistics were calculated using 20 MD simulations remained bound to the active site. Hereafter, we call a state with the ligand SASAs of below and above 50% compared with those of free ligands as a 'deep binding state' and a 'shallow binding state', respectively. The ligand SASAs of free ligands were shown in Supplementary information Table E1. The averages of the energies were calculated from the trajectories of 200-1,000 ns with deep binding states. The minima were also calculated from the same range using the averaged trajectories over 15 (± 7) continuous data points. Figure H1 shows the time courses of MM-GB/SA binding free energies with the same averaging. The lines in faint colours indicate shallow binding states. Figure H2 shows the histograms of MM-GB/SA binding free energies for the trajectories of 200-1,000 ns. The filled boxes and solid lines correspond to the histograms for deep binding states and all states, respectively. The deep binding states had larger binding free energy differences; however, we could not separate deep and shallow bindings clearly because of unimodal distributions. Figure H3 shows the time course of ligand binging sites and MM-GB/SA binding free energies of three 1 μs trajectories with the unbinding events from the active site. The trajectory for darunavir C3 (left) showed the immediate release after binding to the active site around 200 ns, thus the binding was incomplete. The trajectory for nelfinavir B5 (center) showed the gradual increase in the binding free energy after 150 ns and the unbinding occurs at around 220 ns. The trajectory for ritonavir C3 (right) stayed long with the shallow binding state to the active site, and the unbind occurred at around 900 ns.

Supplementary Table H1. List of extended simulations and a summary of the MM-GB/SA free energies. Standard deviations for Δ*G* were shown in parenthesis in the column "Average".

| Ligand | Simulation count | Simulation index | MM-GB/SA Δ*G* (kcal/mol) | |
|---|---|---|---|---|
| | | | Average | Minimum |
| Darunavir | 2 | C6, D7 (C3) | -31.9 (5.6) | -44.1 |
| Indinavir | 3 | C5, C6, D6 | -35.1 (7.7) | -60.6 |
| Lopinavir | 2 | A2, D3 | -38.5 (7.6) | -59.5 |
| Nelfinavir | 3 | B1, C7, D2 (B5) | -34.6(6.8) | -48.6 |
| Ritonavir | 2 | B7, C7 (C3) | -47.6(11.0) | -68.1 |
| Saquinavir | 3 | B1, C4, D3 | -30.0 (5.2) | -46.2 |
| Tipranavir | 5 | A7, B4, B6, C5, D2 | -29.2 (6.5) | -49.5 |

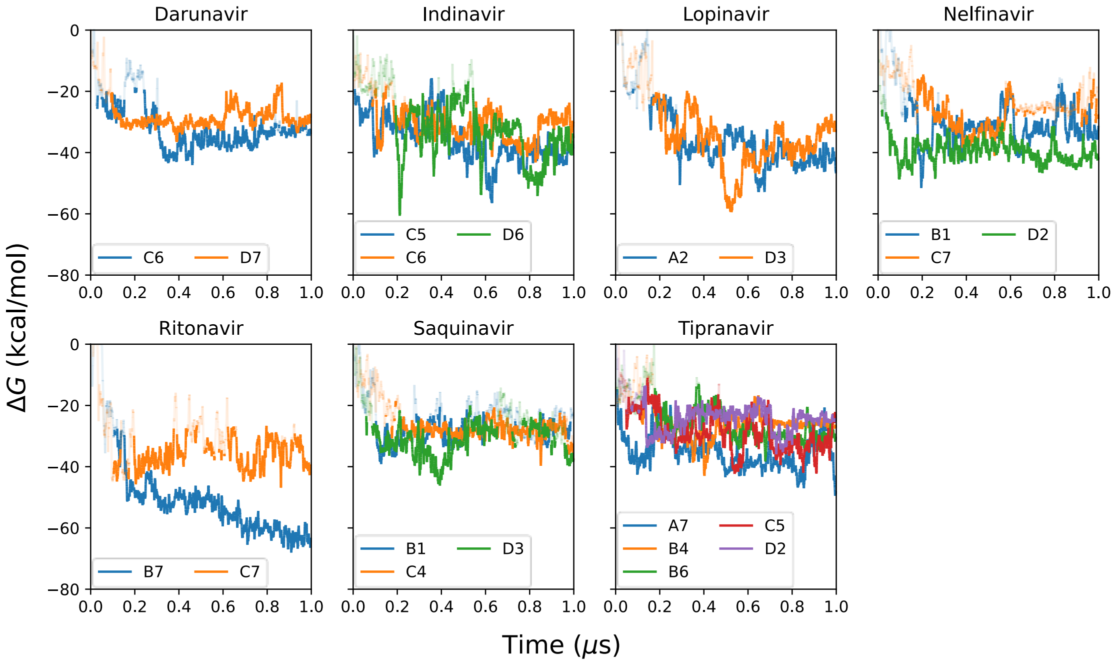


Supplementary Figure H1. Time course of MM-GB/SA binding free energies of 20 extended trajectories of 1 μs.

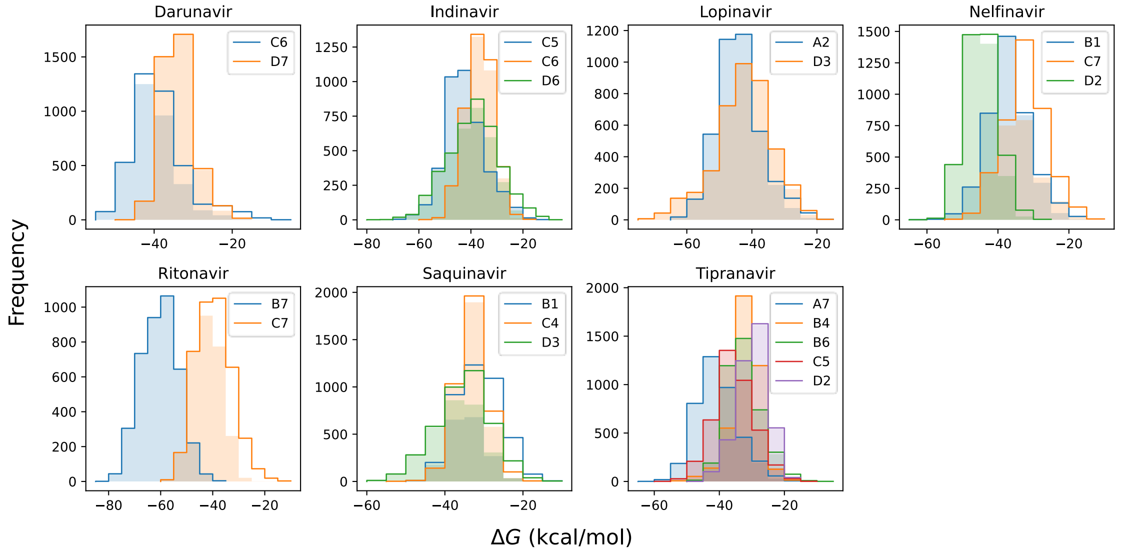


Supplementary Figure H2. Histograms of MM-GB/SA binding free energies of last 800 ns of 1 μs trajectories.

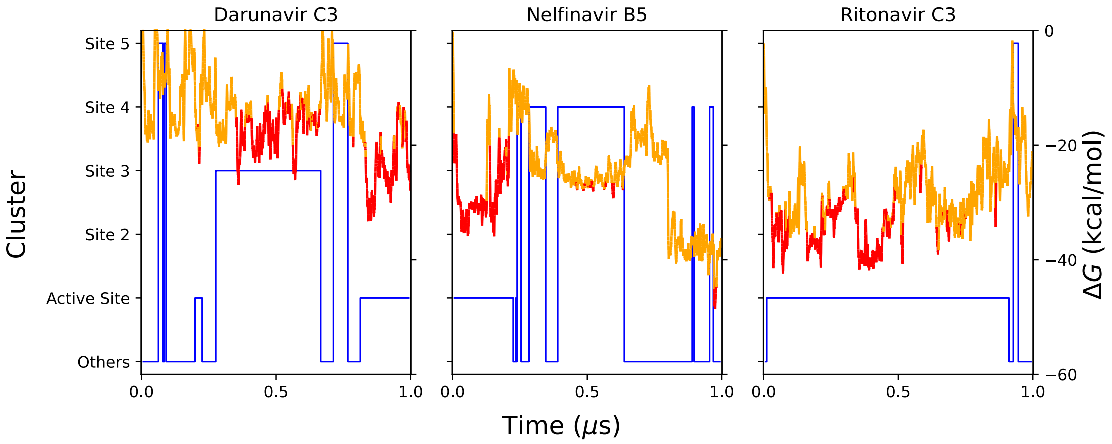


Supplementary Figure H3. Time course of ligand binging sites (blue lines, left axis) and MM-GB/SA binding free energies (red and orange lines, right axis) of three 1 μs trajectories in which the unbinding events from the active site have been observed after 200 ns. The red and orange parts of the lines correspond to the deep and shallow binding states defined by SASA, respectively.

## Supplementary information J. Principal component analysis for characterising the active site conformations. [TOP]

To investigate the conformational variability of the active site upon ligand binding, the principal component analysis (PCA) for Cartesian coordinates of C$\alpha$ atoms of the active site residues were performed by using 20 MD trajectories of ligand bound (holo-M$^{pro}$) system (last 800 ns of each 1 μs long) and a single MD trajectory of ligand-unbound (apo-M$^{pro}$) system (last 1.6 μs of 1.8 μs long). To compare ligand-bound and unbound states effectively, we used 20×400 data points taken every 2 ns from the 20 trajectories of the ligand-bound active sites of the holo-M$^{pro}$ systems and 2×8000 data points taken every 0.2 ns from the trajectory with both active sites of the apo-M$^{pro}$ system. The trajectories of the holo-M$^{pro}$ systems were thinned to balance the number of data points between two states. PCA was performed after superimposing each snapshot of these data points, and the eigenvectors were constructed.

As the target for the inputs of PCA, we selected 37 amino acid residues contained in the active site. Here, we first selected the respective residues that are within 0.45 nm from the inhibitor in the crystallographic structure (PDB ID: 6lu7). In addition, residues 41-54 were added to the defined active site residues because this part is considered to be flexible region from our preliminary result of the MD simulation, and it includes the catalytic residue His41. The residue Leu27 was also added, because it belonged to the active site residues detected by the contact analysis in Supplementary information C (Leu27 has contribution of the mean contact ratio greater than 0.2 to the active site). Taken together, the active site is composed of 37 amino acid residues: residue 24-27 (red), 41-54 (blue), 140-145 (yellow), 163-168 and 172 (green), and 187-192 (magenta) (see Fig. J1). The set of the active site residues defined here includes the whole set of the 25 active site residues classified by the contact cluster analysis in Supplementary Table C2 *clsid*=2 (shown with bold for mean contact ratio greater than 0.2). PCA was calculated using CPPTRAJ[10] module of AmberTools 18.

From the result of PCA, the contribution ratios of first two principal components (PC1 and PC2) were 36.1% and 12.9%, respectively. The first two eigenvectors were shown in Fig. J2. In the projection of the first two principal components, the representative conformations of active site in MD trajectory of apo-M$^{pro}$ system were shown in Fig. J3.

In order to confirm the robustness of the above PCA result, we performed additional PCA by employing the ligand-unbound active site of the holo-M$^{pro}$ system in addition to the ligand-bound active site of the holo-M$^{pro}$ system and the both active sites of the apo-

$M^{pro}$ system used in the above PCA. Here, the trajectories of ligand-bound and unbound active sites of the holo-$M^{pro}$ systems were not thinned; we used the 2×(20×4000) data points taken every 0.2 ns from the 20 trajectories of the ligand-bound and unbound active sites of the holo-$M^{pro}$ systems and 2×8000 data points taken every 0.2 ns from the trajectory with both active sites of apo-$M^{pro}$ system. Similarly, PCA was performed after superimposing each snapshot of these data points, and the eigenvectors were constructed. The obtained set of eigenvectors for PC1 and PC2 was similar to that in the previous PCA result. The projection of PC1 and PC2 was shown in Fig. J4 and their contribution ratios were 34.4% and 12.1%. The projection shown in Fig. 4 looked similar to that in Fig. J4, which indicated that both the PCA gave consistently robust results and captured the characteristic conformational change of the active site of holo- and apo-$M^{pro}$ systems. Since Fig. J4 had denser data points for apo-$M^{pro}$, broader distribution for apo-$M^{pro}$ was observed in Fig. J4 compared to Fig. 4. Of note, rarely visited area in conformational space of the active sites by apo-$M^{pro}$ was more visited by holo-$M^{pro}$.

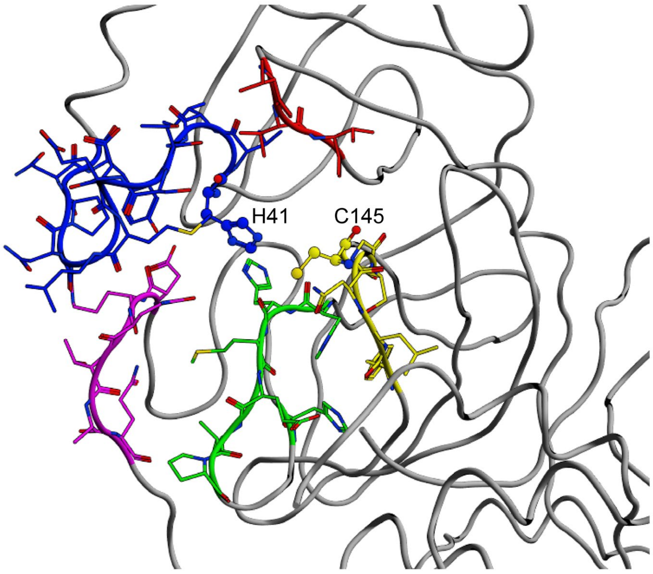


Supplementary Figure J1. The amino acid residue of active site used for PCA. The catalytic dyad, Cys145 and His41, are shown by the ball and stick model. The five regions of the active site, (1) residues 24-27, (2) 41-54, (3) 140-145, (4) 163-168 and 172, and (5) 187-192, are coloured in red, blue, yellow, green, and magenta, respectively.

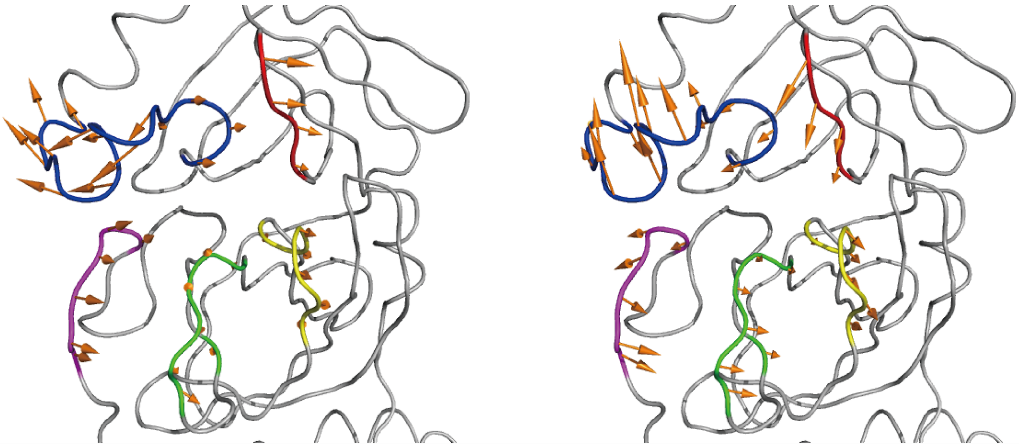

PC1 PC2

Supplementary Figure J2. The protein motion corresponding to the first two eigenvectors obtained from PCA. The arrows indicate the protein motion corresponding to the first two eigenvectors. The five regions of the active site, (1) residues 24-27, (2) 41-54, (3) 140-

145, (4) 163-168 and 172, and (5) 187-192, are coloured in red, blue, yellow, green, and magenta, respectively.

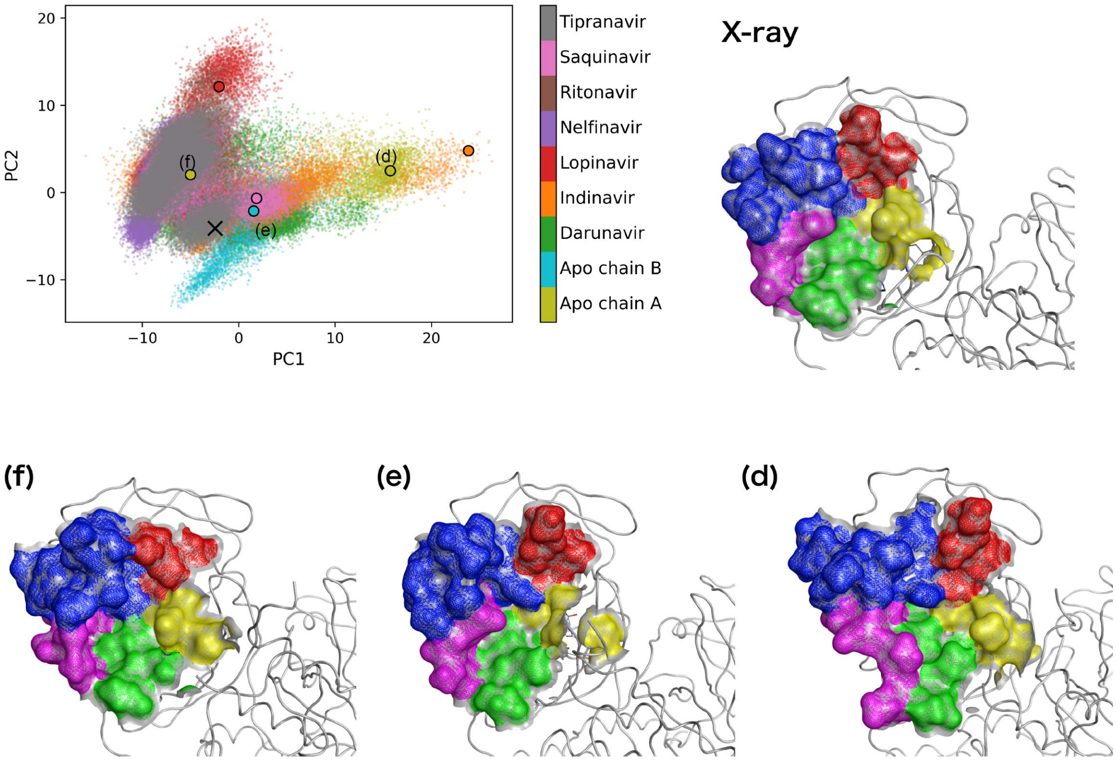


Supplementary Figure J3. Conformational diversity of M$^{pro}$ active site. The projection of the first two principal components (PC1 and PC2) that is the same as that of Fig. 4 are shown. The conformations of the active site are shown for the structures at the cross mark and the points (d)-(f), which correspond to the crystal structure (PDB ID: 6lu7) and simulation structures of apo-M$^{pro}$ systems. The five regions of the active site, (1) residues 24-27, (2) 41-54, (3) 140-145, (4) 163-168 and 172, and (5) 187-192, are coloured in red, blue, yellow, green, and magenta, respectively. In the 2D projection plot, data points were plotted for whole trajectories of the holo and the apo simulations sampled every 0.2 ns, while the axes PC1 and PC2 were constructed by balanced number of data points as described in the text. The Apo chain A and Apo chain B correspond to the trajectories of the active sites of the chain-A and the chain-B of the apo-M$^{pro}$ system, respectively.

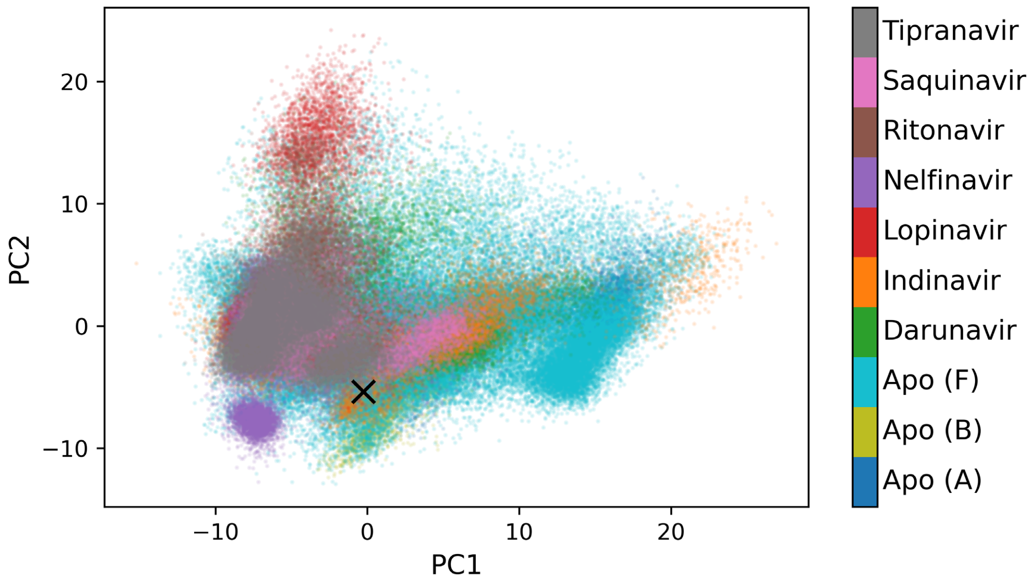


Supplementary Figure J4. The confirmation of the robustness of the PCA result. This figure shows the projection of PC1 and PC2 obtained from PCA using to the 20 trajectories of the ligand-bound and unbound active sites of holo-M$^{pro}$ systems and the trajectory of both active sites of apo-M$^{pro}$ system. The projection shown in Fig. 4 (and the same in Fig. J3) looked similar to this figure, which indicated that both the PCA gave consistently robust results. The cross mark corresponds to the the crystal structure (PDB ID: 6lu7). The Apo (A) and Apo (B) correspond to the trajectory of the active site of chain-A and that of chain-B of the apo-M$^{pro}$ system, respectively. Apo (F) corresponds to the trajectories of ligand-unbound active sites of the holo-M$^{pro}$ systems.

## Supplementary information K. Representative binding poses for MD simulations of respective ligand-bound $M^{pro}$ systems. [TOP]

Here we investigate how each ligand interacted with the active site residues in the MD simulations. In order to detect the effective interaction between the ligand and each residue, the interaction fingerprint was analysed by using the protein-ligand interaction fingerprint (PLIF) tool of Molecular Operating Environment (MOE)[11]. The fingerprint is defined as a set of bits that identify the existence of the ionic or hydrogen bond between the residue atom and the ligand atom with the threshold energy value of 0.5 kcal/mol. The fingerprints were assigned for all snapshots in 20 trajectories of the 1 μs MD simulations. For each ligand, the appearance rates of the bits were given by averages of the fingerprints for the snapshots taken every 2 ns over 200-1000 ns. In order to extract key residues in the protein-ligand interaction, a maximal value of the appearance rates was selected among all bits related for each residue. We here call the value of the maximum appearance rate as the representative appearance rate of the interaction fingerprint (RAIF) that are summarised in Table K1 (see also Fig. K8). The value is expected to reflect an interaction strength between each residue to a specific ligand. Further, in order to pick the representative binding poses, we have performed clustering analysis on the fingerprints using the Jarvis-Patrick clustering algorithm[12] with Tanimoto Superset/Subset (a similarity cutoff value of 60%), and picked a snapshot with the largest count of the interacting residues (among RAIF $\geq$ 1%) with the active bits in PLIF from each of the top three clusters. In case there were multiple snapshots with the largest counts, we selected a pose considering their interaction patterns with some arbitrariness. Figures K1-7 show three representative binding poses for each ligand (purple stick model) selected in this manner, where the side chains within the distance of 0.35 nm from the ligand are plotted with green stick model, the values of RAIF for each ligand are shown by the colour on the surface plot, and the hydrogen bonds are shown with yellow dotted lines.

**Darunavir**

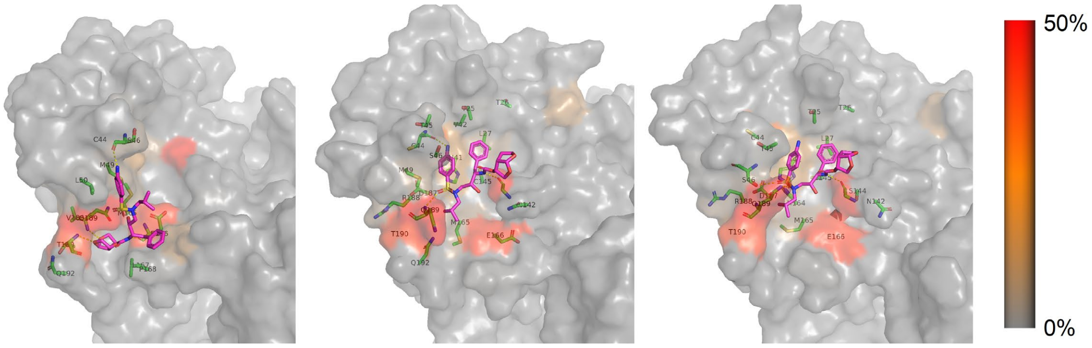


Supplementary Figure K1. The characteristic binding poses for darunavir observed in MD simulations.

**Indinavir**

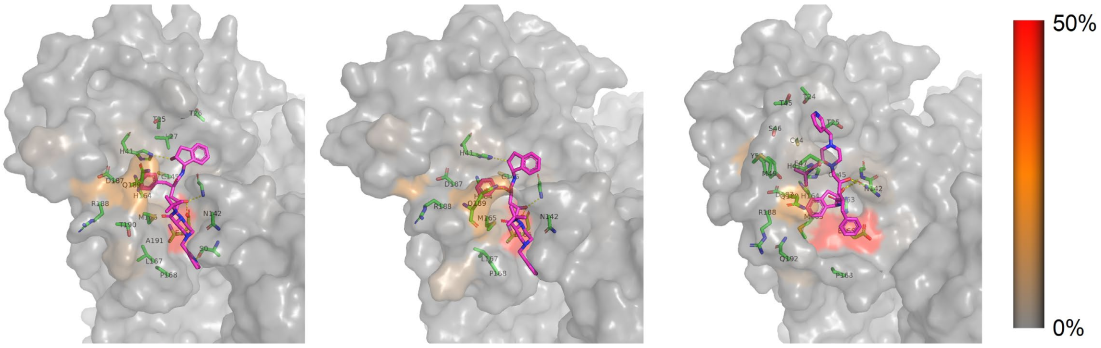


Supplementary Figure K2. The characteristic binding poses for indinavir observed in MD simulations.

**Lopinavir**

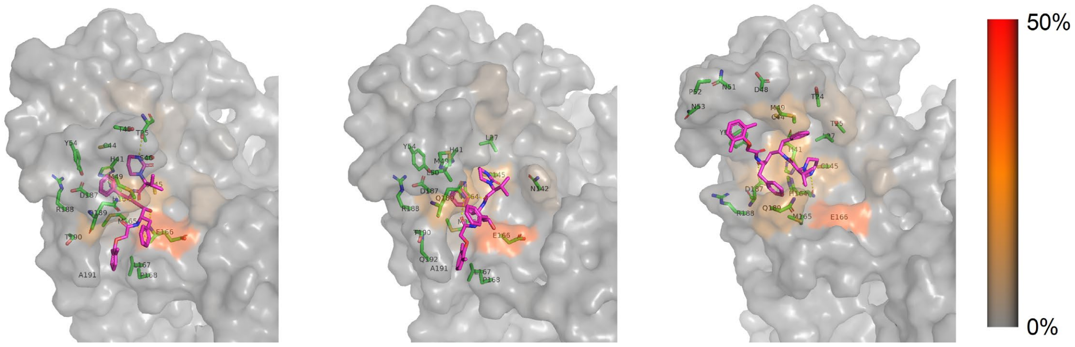


Supplementary Figure K3. The characteristic binding poses for lopinavir observed in MD simulations.

**Nelfinavir**

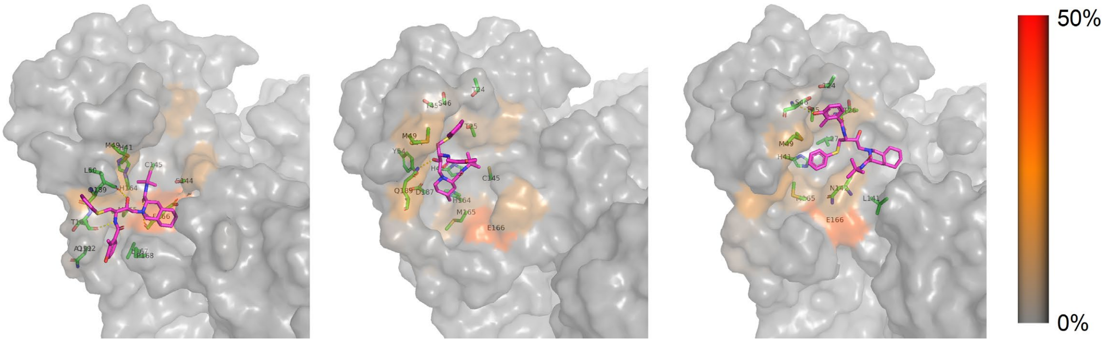


Supplementary Figure K4. The characteristic binding poses for nelfinavir observed in MD simulations.

**Ritonavir**

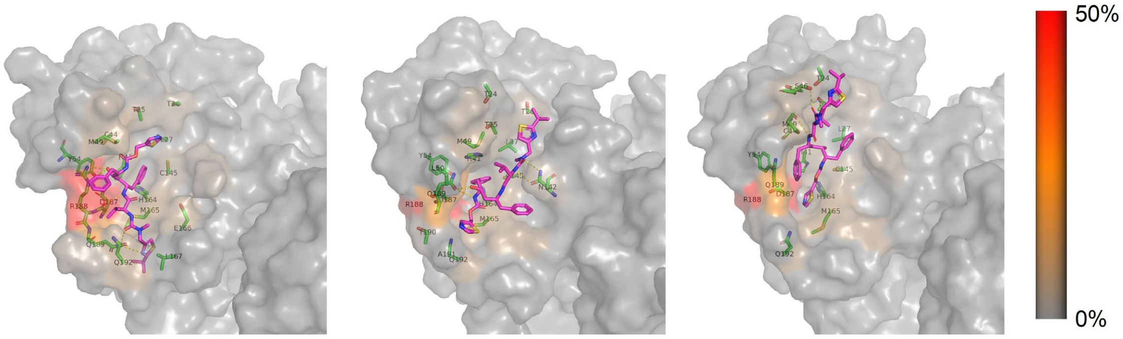


Supplementary Figure K5. The characteristic binding poses for ritonavir observed in MD simulations.

**Saquinavir**

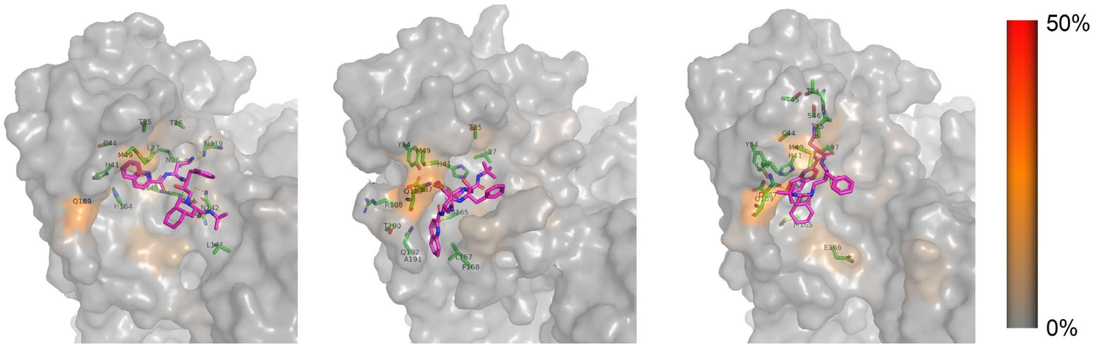


Supplementary Figure K6. The characteristic binding poses for saquinavir observed in MD simulations.

**Tipranavir**

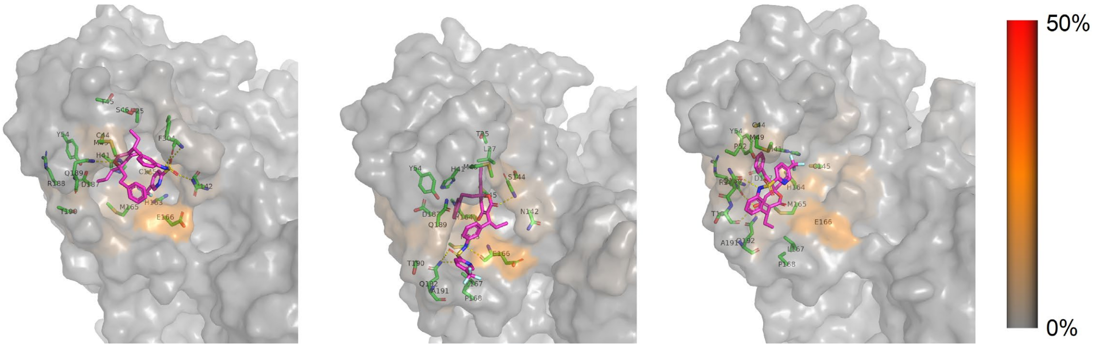


Supplementary Figure K7. The characteristic binding poses for tipranavir observed in MD simulations.

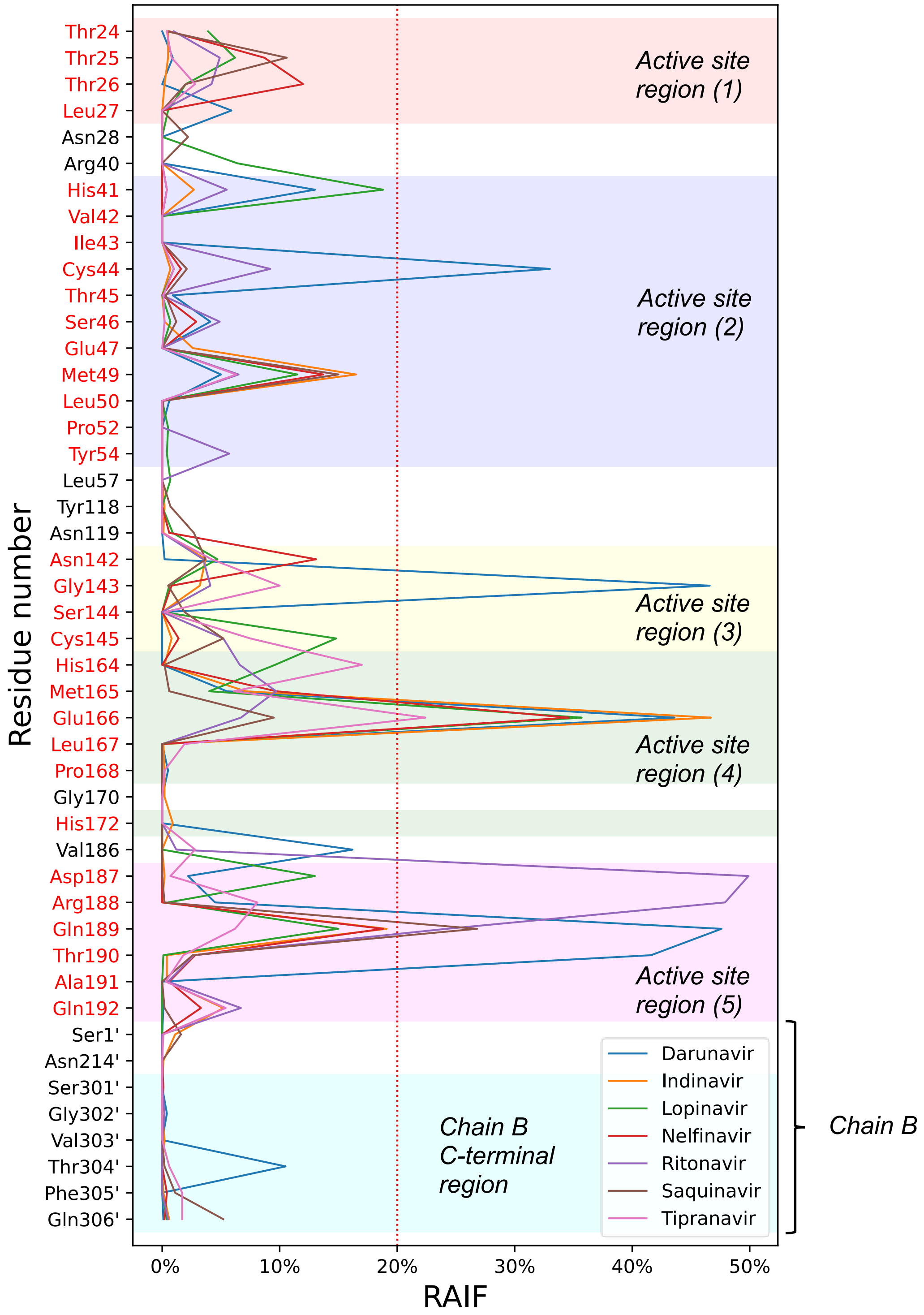


Figure K8. Representative appearance rate of the interaction fingerprint (RAIF) for each residue in the vicinity of the active site (in percent). See also Table K1 for explanations.

Supplementary Table K1. Representative appearance rate of the interaction fingerprint (RAIF) for each residue in the vicinity of the active site (in percent). For this analysis, the active site where the respective ligands bound were regarded to belong to the chain A. The residue numbers without and with prime marks correspond to the chains A and B, respectively. In this table, the amino acid residues that formed the interaction fingerprint at least once in 20 trajectories of the 1 μs MD simulations were listed. The active site residues used in PCA were indicated in red. The columns above 20%, 15%, and 10% are highlighted with yellow, grey, and light grey, respectively.

| ***Residue number*** | ***darunavir*** | ***indinavir*** | ***lopinavir*** | ***nelfinavir*** | ***ritonavir*** | ***saquinavir*** | ***tipranavir*** |
|---|---|---|---|---|---|---|---|
| **Thr24** | 0.0 | 0.5 | 3.9 | 0.6 | 1.0 | 0.4 | 0.4 |
| **Thr25** | 0.9 | 0.5 | 6.2 | 8.7 | 4.9 | 10.6 | 0.8 |
| **Thr26** | 0.0 | 0.2 | 2.0 | 12.0 | 4.2 | 2.0 | 2.8 |
| **Leu27** | 5.9 | 0.0 | 0.5 | 0.0 | 0.0 | 0.0 | 0.0 |
| **Asn28** | 0.0 | 0.0 | 0.0 | 0.0 | 0.0 | 2.2 | 0.0 |
| **Arg40** | 0.0 | 0.0 | 6.4 | 0.0 | 0.0 | 0.0 | 0.0 |
| **His41** | 13.0 | 2.7 | 18.8 | 0.0 | 5.5 | 0.4 | 0.4 |
| **Val42** | 0.0 | 0.0 | 0.0 | 0.0 | 0.0 | 0.0 | 0.0 |
| **Ile43** | 0.0 | 0.0 | 0.0 | 0.0 | 0.0 | 0.0 | 0.0 |
| **Cys44** | **33.0** | 0.7 | 1.0 | 1.6 | 9.2 | 2.1 | 1.0 |
| **Thr45** | 0.9 | 0.0 | 0.0 | 0.0 | 0.0 | 0.3 | 0.1 |
| **Ser46** | 4.1 | 0.2 | 0.7 | 2.9 | 4.9 | 1.2 | 0.2 |
| **Glu47** | 0.0 | 2.6 | 0.0 | 0.1 | 0.0 | 0.0 | 0.0 |
| **Met49** | 5.0 | 16.5 | 11.5 | 13.7 | 6.5 | 15.0 | 6.3 |
| **Leu50** | 0.6 | 0.0 | 0.0 | 0.0 | 0.0 | 0.1 | 0.0 |
| **Pro52** | 0.0 | 0.0 | 0.5 | 0.0 | 0.0 | 0.0 | 0.0 |
| **Tyr54** | 0.0 | 0.0 | 0.4 | 0.0 | 5.7 | 0.0 | 0.0 |
| **Leu57** | 0.0 | 0.0 | 0.7 | 0.0 | 0.0 | 0.0 | 0.0 |
| **Tyr118** | 0.0 | 0.2 | 0.0 | 0.0 | 0.0 | 0.7 | 0.0 |
| **Asn119** | 0.0 | 0.1 | 0.9 | 0.6 | 0.0 | 2.7 | 0.0 |
| **Asn142** | 0.2 | 3.7 | 4.7 | 13.1 | 3.5 | 3.7 | 4.2 |
| **Gly143** | **46.6** | 3.2 | 0.6 | 0.8 | 4.1 | 0.5 | 10.0 |
| **Ser144** | 0.0 | 0.0 | 0.0 | 0.0 | 0.0 | 1.9 | 0.2 |
| **Cys145** | 0.0 | 0.8 | 14.8 | 1.4 | 5.2 | 5.2 | 7.5 |
| **His164** | 0.0 | 0.2 | 9.6 | 0.0 | 6.6 | 0.2 | 17.0 |

| | | | | | | | |
|---|---|---|---|---|---|---|---|
| **Met165** | 5.5 | 7.1 | 4.0 | 9.7 | 9.7 | 0.6 | 6.1 |
| **Glu166** | **43.6** | **46.7** | **35.7** | **34.6** | 6.7 | 9.5 | **22.4** |
| **Leu167** | 0.0 | 0.1 | 0.0 | 0.0 | 0.0 | 0.0 | 1.9 |
| **Pro168** | 0.5 | 0.2 | 0.0 | 0.0 | 0.0 | 0.0 | 0.2 |
| **Gly170** | 0.0 | 0.2 | 0.0 | 0.0 | 0.0 | 0.0 | 0.0 |
| **His172** | 0.0 | 0.9 | 0.0 | 0.0 | 0.0 | 0.0 | 0.0 |
| **Val186** | 16.2 | 0.0 | 0.0 | 0.0 | 1.2 | 0.0 | 2.8 |
| **Asp187** | 2.2 | 0.2 | 13.0 | 0.0 | **49.9** | 0.0 | 0.7 |
| **Arg188** | 4.5 | 0.0 | 0.5 | 0.0 | **47.9** | 0.2 | 8.1 |
| **Gln189** | **47.6** | 19.1 | 15.0 | 18.8 | **24.2** | **26.8** | 6.2 |
| **Thr190** | **41.6** | 0.4 | 0.1 | 2.8 | 2.7 | 2.6 | 1.8 |
| **Ala191** | 0.0 | 0.4 | 0.0 | 0.6 | 0.5 | 0.0 | 0.3 |
| **Gln192** | 0.1 | 5.2 | 0.0 | 3.3 | 6.7 | 0.2 | 5.4 |
| **Ser1’** | 0.0 | 1.1 | 0.0 | 0.0 | 0.0 | 1.6 | 0.1 |
| **Asn214’** | 0.0 | 0.1 | 0.0 | 0.0 | 0.0 | 0.0 | 0.0 |
| **Ser301’** | 0.0 | 0.0 | 0.0 | 0.1 | 0.0 | 0.0 | 0.0 |
| **Gly302’** | 0.4 | 0.0 | 0.0 | 0.0 | 0.2 | 0.0 | 0.0 |
| **Val303’** | 0.0 | 0.2 | 0.0 | 0.0 | 0.0 | 0.0 | 0.0 |
| **Thr304’** | 10.5 | 0.0 | 0.0 | 0.0 | 0.0 | 0.2 | 0.6 |
| **Phe305’** | 0.0 | 0.1 | 0.0 | 0.4 | 0.0 | 1.1 | 1.7 |
| **Gln306’** | 0.1 | 0.6 | 0.4 | 0.2 | 0.5 | 5.2 | 1.7 |

## Supplementary information L. Molecular docking using the X-ray crystal and MD simulation structures. [TOP]

To figure the feature of the active site conformations sampled by the MD simulations, we performed the conventional molecular docking using the four protein structures (one derived from X-ray crystallographic and three from representative MD simulations shown in Figure 4) and the drug-like compound library. All molecular dockings were performed using Glide module[13]–[15], implemented in the Schrödinger Release 2020-1[16]. The conditions for molecular docking were as follows. For generating receptor grids for the docking, a side length of cubic grids was set to 10 Å and no constraints were applied. The grid center was set at an arbitrary point near two catalytic residues, His41 and Cys145. To soften the potential of nonpolar parts of ligands, the scaling factor for the ligand van der Waals radii was set in 0.80. The OPLS3 force field was used[17]. All compounds were docked into the active site of $M^{pro}$ using Glide standard precision (SP) mode. For the compound library, about 14,000 drug-like compounds contained in Maybridge HitCreator V2 were used[18]. The compound molecules were prepared using LigPrep module, implemented in the Schrödinger Release 2020-1[19]. Hydrogen atoms were added and different protonation states and ionization states for each ligand were generated for a pH of 7. All possible stereoisomers and tautomeric states were also generated. The OPLS3 force field[17] was used for energy minimization to generate low energy three dimensional conformers of the ligands.

Table L1 shows the overlap ratio between each pair of top scored compound sets obtained with four different protein structures. Upper and lower triangular portions show the overlaps for the top 100 and 1,000 scored compound sets, respectively. There were only 10 % or less overlap in any two sets of all the top 100 scored sets and about 20 % overlap in any two sets of all the top 1,000 scored sets. This result indicated that the distinct set of promising inhibitor candidates was obtained by molecular dockings based on each employed protein structure sampled by the MD simulations. This is supported by the fact that these active site conformations were very different at protein backbone level (described in the results section on "Conformational variations upon ligand binding" and Fig. 4). Moreover, it suggests that the ensemble docking approach using multiple protein structures rather than the conventional docking using a single protein structure is suited for the $M^{pro}$ system.

Supplementary Table L1. Overlap ratios (in percent) of the top 100 (upper triangular) and the top 1,000 (lower triangular) scored compound sets. Crystal structure, indinavir-bound $M^{pro}$, saquinavir-bound $M^{pro}$, and lopinavir-bound $M^{pro}$ corresponded to the protein structures of X-ray, (a), (b), and (c) in Fig. 4, respectively.

| Protein structure | crystal structure | indinavir-bound $M^{pro}$ | saquinavir-bound $M^{pro}$ | lopinavir-bound $M^{pro}$ |
|---|---|---|---|---|
| crystal structure | | 8.0 | 6.0 | 2.0 |
| indinavir-bound $M^{pro}$ | 21.7 | | 10.0 | 5.0 |
| saquinavir-bound $M^{pro}$ | 19.6 | 22.8 | | 6.0 |
| lopinavir-bound $M^{pro}$ | 20.6 | 23.2 | 23.2 | |

## Supplementary information M. Binding pose flipping over long time scale of MD trajectories.

[TOP]

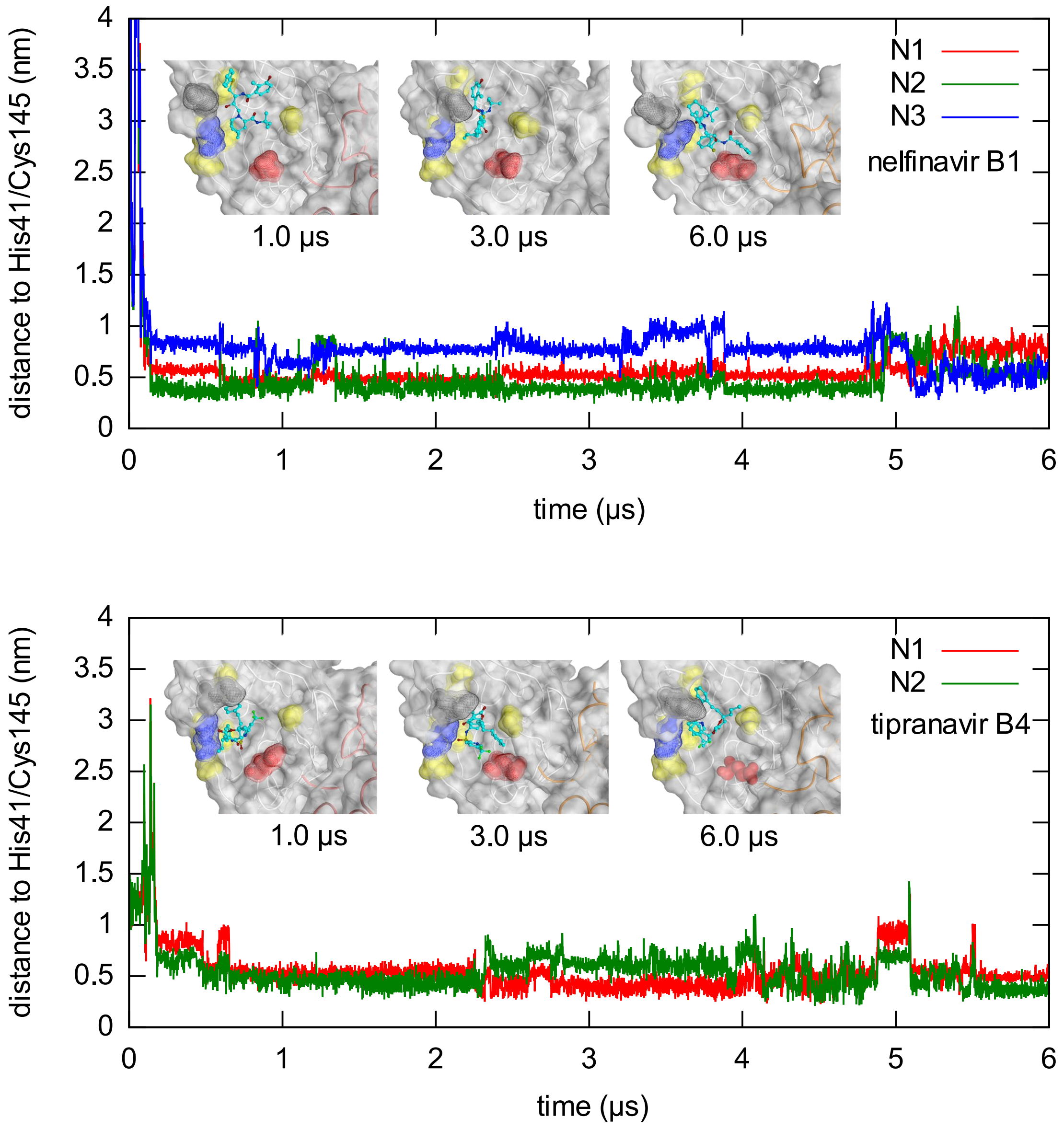


Supplementary Figure M1. Ligand flipping during 6 µs MD simulations. For each nitrogen atom in the ligand, minimum distance to the catalytic residues His41/Cys145 is plotted every 1 ns. These distances reflect binding pose of the ligand, and thus flipping events are visualised as a sudden switching of these distances. Inset shows ligand poses at 1.0, 3.0, and 6.0 µs, where surfaces of the active site are coloured as in Fig. 5.

## Supplementary information N. Interaction of the C-terminal residue of the other chain of the dimer with ligands and the drug binding site.

[TOP]

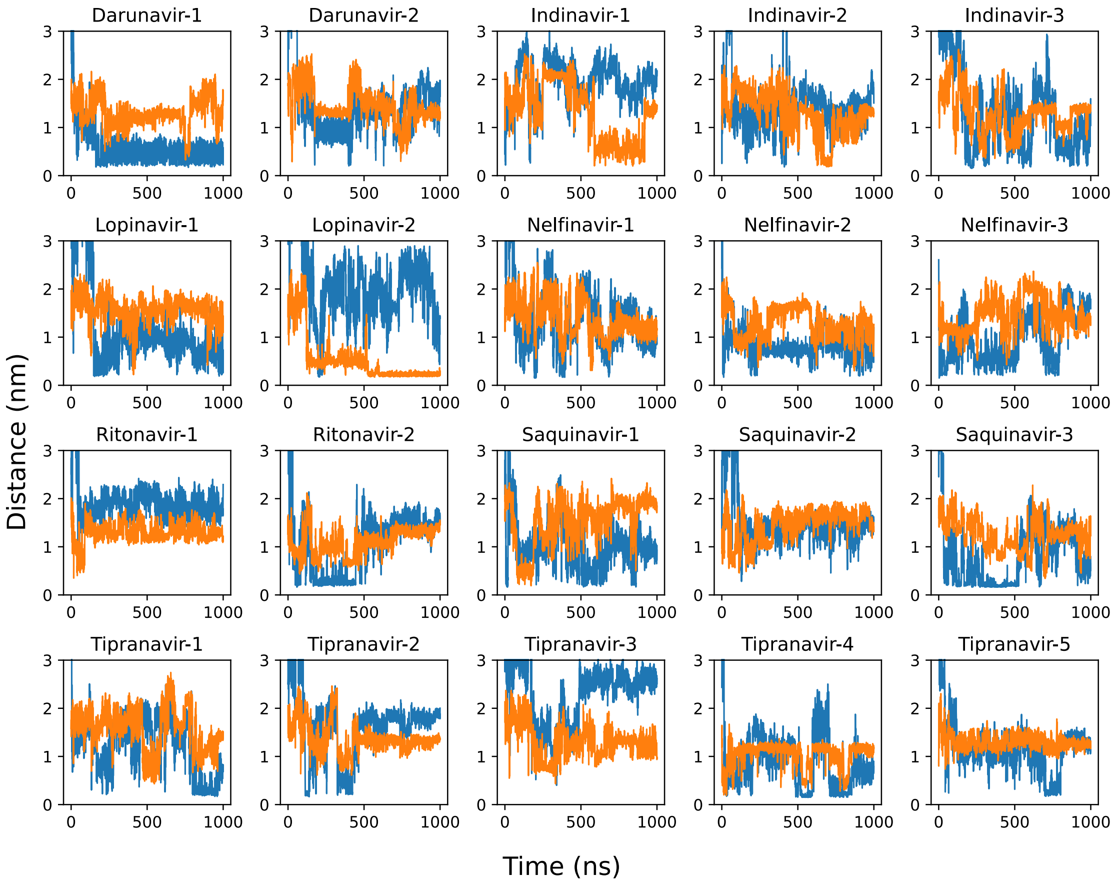


Supplementary Figure N1. Time series of the minimum distances from the C-terminal residue Gln306 of the chain B to the ligands (blue) and to the active site residue Cys145 of the chain A (orange). The chain A is defined as the chain with the active site with ligands, and the chain B is another chain in the dimer.

Supplementary Table N1. Fraction of time that the C-terminal residue Gln306 of the chain B stayed within 0.35 nm of the ligands and the active site residue Cys145 of the chain A. Here, the chain A is defined as the chain with the active site with ligands, and the chain B is another chain in the dimer. The minimum distances were measured.

| | To Ligand | To Cys145 |
|---|---|---|
| Darunavir | 0.087 | 0.000 |
| Indinavir | 0.029 | 0.028 |
| Lopinavir | 0.027 | 0.293 |
| Nelfinavir | 0.033 | 0.000 |
| Ritonavir | 0.138 | 0.000 |
| Saquinavir | 0.132 | 0.000 |
| Tipranavir | 0.107 | 0.001 |
| Average | 0.081 | 0.034 |

## Supplementary information Y. Summary of numerical methods.

[TOP]

Supplementary Table Y1. Summary of numerical methods.

| | | |
|---|---|---|
| Machine | Intel PC cluster (HOKUSAI Big Waterfall / Sailing Ship system) | Special-purpose computer MDGRAPE-4A |
| Software | GROMACS[1] 2020.1/2018.8 | Inhouse code, partly derived from GROMACS[1] |
| Long range Coulomb | SPME[20] | TME (see Supplementary information Z) |
| Constraint | LINCS[21](h-bonds) SETTLE[22](TIP3P) | RATTLE[23](h-bonds) SETTLE[22](TIP3P) |
| Thermostat | the canonical sampling velocity rescaling (CSVR)[24] | Same as left |

## Supplementary information Z. Computation of Coulomb forces for MDGRAPE-4A. [TOP]

For MD simulations by MDGRAPE-4A, Coulomb forces were computed by tensor-structured multilevel Ewald summation method (TME) (Y. M. Koyama et al., in preparation). TME can be considered as a combination of the smooth particle mesh Ewald method (SPME)[20] and the B-spline multilevel summation method (B-spline MSM)[25] with approximations by Gaussian functions. In the current study, the Coulomb potential $g(r) \coloneqq 1/r = \left(2/\sqrt{\pi}\right) \int_0^\infty \exp(-u^2 r^2)\, \mathrm{d}u$ is divided into three part: short $g_\mathrm{S}(r) := \left(2/\sqrt{\pi}\right) \int_\alpha^\infty \exp(-u^2 r^2)\, \mathrm{d}u$, middle $g_\mathrm{M}(r) := \left(2/\sqrt{\pi}\right) \int_{\alpha/s}^\alpha \exp(-u^2 r^2)\, \mathrm{d}u$, and long-range part $g_\mathrm{L}(r) := \left(2/\sqrt{\pi}\right) \int_0^{\alpha/s} \exp(-u^2 r^2)\, \mathrm{d}u$ with the scaling factor $s = 2$ and the Ewald splitting parameter $\alpha = 2.116203\ \mathrm{nm}^{-1}$ (corresponding to *ewald-rtol* = $10^{-4}$ in GROMACS[1]). Since the short-range part $g_\mathrm{S}(r)$ is identical to one in SPME, we computed them by the direct summation with cutoff distance 1.3 nm. The middle and long-range part is evaluated by the combination of long-range part of SPME and B-spline MSM with 32 grids for each axis and 6 order B-spline interpolation. To approximate the middle-range part $g_\mathrm{M}(r)$ with Gaussian functions, we used 4-points Gauss-Legendre quadrature. The approximation by Gaussian functions enables the computation of the 3D convolution to convert grid charges into the grid potentials by separable convolutions[26]. The long-range part can be computed similarly to the one of SPME with the long-range potential $g_\mathrm{L}(r)$, which uses the fast Fourier transformation for the computation of grid potentials from grid charges. Due to the scaling factor $s = 2$, the long-range part was evaluated with 16 grids for each axis. Conversions from $32 \times 32 \times 32$ to $16 \times 16 \times 16$ grid charges (called restriction) and from $16 \times 16 \times 16$ to $32 \times 32 \times 32$ grid potentials (called prolongation) can also be computed by separable convolutions[25].

## References in Supplementary information [TOP]


[1] Abraham, M. J. *et al.* Gromacs: High performance molecular simulations through multi-level parallelism from laptops to supercomputers. *SoftwareX* (2015) doi:10.1016/j.softx.2015.06.001.

[2] Ward, J. H. Hierarchical Grouping to Optimize an Objective Function. *J. Am. Stat. Assoc.* (1963) doi:10.2307/2282967.

[3] Müllner, D. fastcluster : Fast Hierarchical , Agglomerative. *J. Stat. Softw.* (2013) doi:10.18637/jss.v053.i09.

[4] Hartigan, A. & Wong, M. A. A K-Means Clustering Algorithm. *J. R. Stat. Soc.* (1979) doi:10.2307/2346830.

[5] R Core Team. A Language and Environment for Statistical Computing. *R Foundation for Statistical Computing, Vienna, Austria. URL http://www.R-project.org/.* (2019).

[6] Miller, R. G. Jackknifing Variances. *Ann. Math. Stat.* (1968) doi:10.1214/aoms/1177698418.

[7] Miller, B. R. *et al.* MMPBSA.py: An efficient program for end-state free energy calculations. *J. Chem. Theory Comput.* (2012) doi:10.1021/ct300418h.

[8] Onufriev, A., Bashford, D. & Case, D. A. Exploring Protein Native States and Large-Scale Conformational Changes with a Modified Generalized Born Model. *Proteins Struct. Funct. Genet.* (2004) doi:10.1002/prot.20033.

[9] Weiser, J., Shenkin, P. S. & Still, W. C. Approximate solvent-accessible surface areas from tetrahedrally directed neighbor densities. *Biopolymers* (1999) doi:10.1002/(SICI)1097-0282(19991005)50:4<373::AID-BIP3>3.0.CO;2-U.

[10] Roe, D. R. & Cheatham, T. E. PTRAJ and CPPTRAJ: Software for processing and analysis of molecular dynamics trajectory data. *J. Chem. Theory Comput.* (2013) doi:10.1021/ct400341p.

[11] ULC, C. C. G. Molecular Operating Environment (MOE), 2013.08. *1010 Sherbooke St. West, Suite #910, Montreal, QC, Canada, H3A 2R7* (2018) doi:10.1038/ja.2010.129.

[12] Jarvis, R. A. & Patrick, E. A. Clustering Using a Similarity Measure Based on Shared Near Neighbors. *IEEE Trans. Comput.* (1973) doi:10.1109/T-C.1973.223640.

[13] Halgren, T. A. *et al.* Glide: A New Approach for Rapid, Accurate Docking and Scoring. 2. Enrichment Factors in Database Screening. *J. Med. Chem.* (2004) doi:10.1021/jm030644s.

[14] Friesner, R. A. *et al.* Glide: A New Approach for Rapid, Accurate Docking and Scoring. 1. Method and Assessment of Docking Accuracy. *J. Med. Chem.* (2004) doi:10.1021/jm0306430.

[15] Friesner, R. A. *et al.* Extra precision glide: Docking and scoring incorporating a model of hydrophobic enclosure for protein-ligand complexes. *J. Med. Chem.* (2006) doi:10.1021/jm051256o.

[16] Schrödinger. Glide. *Schrödinger Release 2020-2 Schrödinger, LLC, New York, NY, 2020.* (2020).

[17] Harder, E. *et al.* OPLS3: A Force Field Providing Broad Coverage of Drug-like Small Molecules and Proteins. *J. Chem. Theory Comput.* (2016) doi:10.1021/acs.jctc.5b00864.

[18] Maybridge. HitCreator. https://www.maybridge.com/.

[19] Schrödinger. LigPrep. *Schrödinger Release 2020-1: Schrödinger, LLC, New York, NY, 2020* (2020).

[20] Essmann, U. *et al.* A smooth particle mesh Ewald method. *J. Chem. Phys.* (1995) doi:10.1063/1.470117.

[21] Hess, B., Bekker, H., Berendsen, H. J. C. & Fraaije, J. G. E. M. LINCS: A Linear Constraint Solver for molecular simulations. *J. Comput. Chem.* (1997) doi:10.1002/(SICI)1096-987X(199709)18:12<1463::AID-JCC4>3.0.CO;2-H.

[22] Miyamoto, S. & Kollman, P. A. Settle: An analytical version of the SHAKE and RATTLE algorithm for rigid water models. *J. Comput. Chem.* (1992) doi:10.1002/jcc.540130805.

[23] Andersen, H. C. Rattle: A 'velocity' version of the shake algorithm for molecular dynamics calculations. *J. Comput. Phys.* (1983) doi:10.1016/0021-9991(83)90014-1.

[24] Bussi, G., Donadio, D. & Parrinello, M. Canonical sampling through velocity rescaling. *J. Chem. Phys.* (2007) doi:10.1063/1.2408420.

[25] Hardy, D. J., Wolff, M. A., Xia, J., Schulten, K. & Skeel, R. D. Multilevel summation with B-spline interpolation for pairwise interactions in molecular dynamics simulations. *J. Chem. Phys.* (2016) doi:10.1063/1.4943868.

[26] Shaw, D. E. *et al.* Anton 2: Raising the Bar for Performance and Programmability in a Special-Purpose Molecular Dynamics Supercomputer. in *International Conference for High Performance Computing, Networking, Storage and Analysis, SC* (2014). doi:10.1109/SC.2014.9.
